# Functional Analysis of Host Factors that Mediate the Intracellular Lifestyle of *Cryptococcus neoformans*

**DOI:** 10.1371/journal.ppat.1002078

**Published:** 2011-06-16

**Authors:** Qing-Ming Qin, Jijing Luo, Xiaorong Lin, Jianwu Pei, Lei Li, Thomas A. Ficht, Paul de Figueiredo

**Affiliations:** 1 Department of Plant Pathology and Microbiology, Texas A&M University, College Station, Texas, United States of America; 2 Borlaug Advanced Research Center, Texas A&M University, College Station, Texas, United States of America; 3 Department of Biology, Texas A&M University, College Station, Texas, United States of America; 4 Department of Veterinary Pathobiology, Texas A&M University, College Station, Texas, United States of America; University of Melbourne, Australia

## Abstract

*Cryptococcus neoformans* (Cn), the major causative agent of human fungal meningoencephalitis, replicates within phagolysosomes of infected host cells. Despite more than a half-century of investigation into host-Cn interactions, host factors that mediate infection by this fungal pathogen remain obscure. Here, we describe the development of a system that employs *Drosophila* S2 cells and RNA interference (RNAi) to define and characterize Cn host factors. The system recapitulated salient aspects of fungal interactions with mammalian cells, including phagocytosis, intracellular trafficking, replication, cell-to-cell spread and escape of the pathogen from host cells. Fifty-seven evolutionarily conserved host factors were identified using this system, including 29 factors that had not been previously implicated in mediating fungal pathogenesis. Subsequent analysis indicated that Cn exploits host actin cytoskeletal elements, cell surface signaling molecules, and vesicle-mediated transport proteins to establish a replicative niche. Several host molecules known to be associated with autophagy (Atg), including Atg2, Atg5, Atg9 and Pi3K59F (a class III PI3-kinase) were also uncovered in our screen. Small interfering RNA (siRNA) mediated depletion of these autophagy proteins in murine RAW264.7 macrophages demonstrated their requirement during Cn infection, thereby validating findings obtained using the *Drosophila* S2 cell system. Immunofluorescence confocal microscopy analyses demonstrated that Atg5, LC3, Atg9a were recruited to the vicinity of Cn containing vacuoles (CnCvs) in the early stages of Cn infection. Pharmacological inhibition of autophagy and/or PI3-kinase activity further demonstrated a requirement for autophagy associated host proteins in supporting infection of mammalian cells by Cn. Finally, systematic trafficking studies indicated that CnCVs associated with Atg proteins, including Atg5, Atg9a and LC3, during trafficking to a terminal intracellular compartment that was decorated with the lysosomal markers LAMP-1 and cathepsin D. Our findings validate the utility of the *Drosophila* S2 cell system as a functional genomic platform for identifying and characterizing host factors that mediate fungal intracellular replication. Our results also support a model in which host Atg proteins mediate Cn intracellular trafficking and replication.

## Author Summary

*Cryptococcus neoformans* is a facultative intracellular fungal pathogen that causes cryptococcosis in both immunocompromised and immunocompetent individuals worldwide. Initial infection usually occurs in the lungs, but the fungus can disseminate to other organs. The pathogen shows a predilection to the central nervous system (CNS), which can result in life-threatening cryptococcal meningoencephalitis. Significant progress has been made in developing molecular genetic approaches to elucidate mechanisms of Cn pathogenesis. However, because of the unavailability of genetically tractable host cell systems, host factors that mediate the phagocytosis, intracellular replication and escape of Cn have remained obscure. Our data demonstrate that the combination of *Drosophila* S2 cells and RNA interference technology provides a powerful platform for identifying and characterizing host factors that mediate Cn infection. After screening over 400 genes that were annotated to be associated with host cell membrane trafficking and phagosome formation, we identified 57 evolutionarily conserved gene products that when depleted significantly altered the infection phenotype of the pathogen. Finally, we demonstrated that Cn infection of host cells requires autophagy proteins (including Atg2a, Atg5 and Atg9a) and class III PI3-kinase activities, thereby implicating host cell autophagy in supporting the intracellular lifestyle of the pathogen. Our work contributes to understanding host mechanisms that mediate the intracellular survival and dissemination of Cn.

## Introduction

Over the past half-century, *Cryptococcus neoformans* (Cn), an opportunistic encapsulated yeast, has emerged as the major causative agent of fungal meningoencephalitis in humans and animals worldwide [1], [2]. The pathogen is found in a variety of environmental niches, including soil, vegetation, and avian excreta. Initial infection in the lungs results from the inhalation of fungal cells from the environment [1], [2]. The most common clinical form of systemic *Cryptococcus* infection in humans is meningoencephalitis.

An elucidation of the molecular mechanisms mediating the interaction of Cn with host cells will further the development of measures to control cryptococcosis [3]. Several Cn features, such as polysaccharide capsule synthesis [4], [5], melanin production [6], [7], and growth at host physiological temperatures [7] are associated with virulence. In addition, several signaling pathways, including the cAMP-PKA pathway, three MAP kinase pathways (involving Cpk1, Hog1 and Mpk1), the Ras specific pathway, and the Ca^2+^-calcineurin pathway modulate Cn morphological differentiation, virulence, and stress responses [8]. Numerous Cn virulence factors have also been characterized [9].

Insights into host cell functions that mediate infection have also been garnered. For example, host toll-like receptors (TLRs) help recognize fungal pathogens such as *Candida albicans*, *Aspergillus fumigatus* and Cn [10]. Recognition of pathogen-associated molecular patterns (PAMPs) by TLRs, either alone or with other TLR or non-TLR receptors, induces signals responsible for activation of the host innate immune response [10]. Recent studies have demonstrated that MyD88, but neither TLR2 nor TLR4, plays a central role in host defense against Cn [11], [12]. A variety of host cells, such as T helper type 1 (Th1) [13], B cells [14], [15], gamma-delta antigen receptor-bearing T (γ δ T) cells [15], dendritic cells [16], alveolar macrophages [17] and bronchial epithelial cells [18] are known to be involved in coordinating the host immune response to Cn infection. Cell-mediated host immunity to infection is characterized by immune cell activation and secretion of cytokines, including gamma interferon (IFN-γ), IL-12, IL-18, IL-23 and tumor necrosis factor alpha (TNF-α) [1]. Despite these insights, the molecular mechanisms mediating host cell invasion, intracellular replication and escape of Cn from host cells remain obscure.

The fate of internalized Cn cells is cell type-dependent. However, common patterns of intracellular trafficking are observed. After entry into host cells, Cn resides in early endosomal antigen 1 (EEA1)-positive compartments [16], and also interacts with membranes containing the endosome/lysosome markers CD63 [19] and lysosomal-associated membrane protein 1 (LAMP-1) [16], [20], [21]. The pathogen is killed in the lysosomes of dendritic cells [16]. However, in macrophages, Cn cells can survive, replicate, and extrude from phagosome/phagolysosome compartments without inducing host cell death [21], [22]. This extrusion of replicative Cn cells may constitute a mechanism for disseminating Cn among host cells or tissues while limiting detection by host surveillance systems [21], [23]. Despite these observations, the subcellular pathways that Cn uses to enter, replicate and escape from host cells remain obscure.

Recent studies have indicated that activation of the host cell autophagy pathway can control the replication of intracellular pathogens [24], [25], [26]. Autophagy is a highly conserved process in which cellular materials, including proteins and organelles, are engulfed into an autophagosome, a specialized double membrane bounded compartment that delivers material to lysosomes for degradation [27]. Nutrient starvation, endoplasmic reticulum stress, and pathogen attack and/or subversion can activate the autophagy machinery. Autophagosome biogenesis requires the activity of autophagy-specific proteins, including Atg5, an ubiquitin-like protein. Atg5 regulates the conversion of microtubule-associated protein 1 light chain 3 (LC3-I) to a lipidated form LC3-II, which localizes to the autophagic membrane [28], [29]. Membrane-bound LC3-II interacts with the adaptor molecules p62 and NBR1, which capture cytoplasmic cargo earmarked for autophagic degradation [26]. Therefore, the amount of LC3-II within a cell reflects the amount of newly formed autophagic membrane and/or the number of autophagosomes.

Previous studies have demonstrated that host autophagy may be involved in inhibiting the growth of intracellular pathogens, including intracellular bacteria [30], [31], [32] and parasites [32], [33]. However, some microorganisms, including bacteria such as *Porphyromonas gingivalis*, *Brucella abortus*, and *Coxiella burnetii* [34], and viruses [35], [36], [37] may subvert host autophagic processes to promote their intracellular survival and replication.

We exploited *Drosophila melanogaster* S2 cells and RNAi technology to identify host factors that mediate the phagocytosis, intracellular replication and escape of Cn from host cells. Several features of the *Drosophila* cell system made it an attractive choice for these studies. First, the macrophage-like *Drosophila* S2 cell system has proven useful for elucidating evolutionarily conserved components of innate immunity [38], [39]. Second, RNAi strategies can be used to efficiently deplete proteins in these cells. Third, S2 cells have been successfully employed to identify host factors for several microbial pathogens, including *Listeria monocytogenes* [38], [40], *Brucella melitensis* [41] and the fungal pathogen *C. albicans* [39]. Importantly, findings in insect cell models have been validated in their mammalian cell counterparts [41], [42], [43], [44]. Thus, the insect cell system has proven useful for resolving physiologically relevant host factors in evolutionarily divergent organisms. Finally, some *Cryptococcus* species have been shown to reside in a variety of environmental niches, including in association with insects [45], [46]. Moreover, both the caterpillar *Galleria mellonella* [47] and *D. melanogaster* [48] have been previously used as model hosts to study fungal virulence and host defense. Therefore, *Drosophila* cells constitute a host system from which biologically relevant information can be extracted.

Here, we demonstrate that *Drosophila* S2 cells share striking similarities with their mammalian host cell counterparts in supporting the uptake, intracellular replication, cell-to-cell dissemination and escape of Cn cells. We also demonstrate that Cn interacts with host cell membranes containing the autophagosome marker LC3, the endosome/lysosome marker LAMP-1, and the lysosome marker cathepsin D in *Drosophila* S2 cells, murine J774.A1 and RAW264.7 macrophages and mouse embryonic fibroblasts (MEFs). Finally, we demonstrate that several autophagy-associated proteins mediate Cn infection in these systems, thereby implicating the autophagy pathway in supporting the intracellular lifestyle of the pathogen. These data provide the first functional genomic analysis of Cn host factors and provide new insights into mechanisms mediating this host-pathogen interaction.

## Results

### *Drosophila* S2 cells support Cn infection

**Figure 1:**
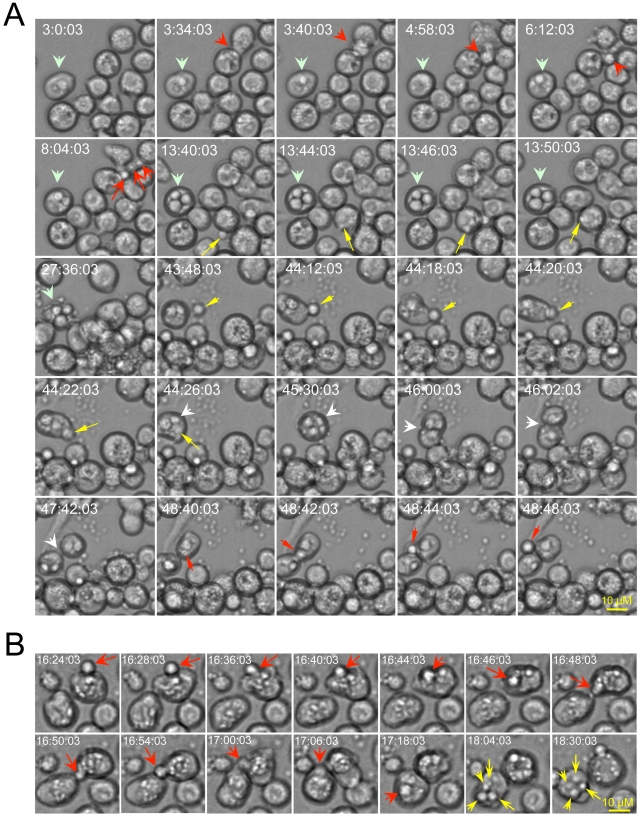
Live cell images indicate that *Drosophila* S2 cells support the phagocytosis, intracellular replication, cell-to-cell dissemination and escape of *Cryptococcus neoformans* (Cn). Live cell images were selected from a representative time-lapse movie (n = 3). Time-lapse images were taken every 2 min. Frames are labeled according to the start of the imaging process, which was initiated approximately 3.5 hrs post-infection. Before the imaging was initiated, the infected cells were washed three times with 1×PBS. Fresh serum-free medium supplemented with 20 µg/ml fluconazole was then added to the cells to inhibit the replication of the extracellular (but not intracellular) population of Cn cells. **A**. *Drosophila* S2 cells support Cn infection. Yellow arrows: Phagocytosis of Cn cells by S2 cells. Light green arrows: Cn replication precedes host cell lysis. Red arrows: Examples of Cn extrusion from host cells. White arrow: Cn cell-to-cell dissemination by host cell division. **B**. Cn direct cell-to-cell spread (red arrows) and intracellular replication (yellow arrows) in S2 cells.

Time-lapse microscopy of live cells provides a compelling approach for visualizing dynamic interactions between host and pathogen cells. Moreover, this approach has proven useful for demonstrating extrusion of Cn from host cells [21], [22]. We employed time-lapse microscopy to determine whether *Drosophila* S2 cells can support infection by Cn. An analysis of time-lapse images revealed several important aspects of this interaction. First, S2 cells efficiently internalized Cn from the surrounding medium (Figure 1A, yellow arrows; Supplemental Videos 1 and 2 at: http://www.youtube.com/user/deFigueiredoLab). Second, internalized Cn cells were observed to replicate within S2 cells (Figure 1A, wide light-green arrows; Figure 1B, yellow arrows; Supplemental Video 1) with a doubling time of ∼2 to 3 hrs. Third, Cn cells were observed to extrude from host cells, either in groups or individually (Figure 1A, red arrows and Supplemental Video 1 and 2). Cn extrusion across the plasma membrane was rapid (<2 min) and was not accompanied by host cell lysis (Figure 1A, Supplemental Videos 1 and 2). Finally, Cn cell-to-cell dissemination by means of re-uptake of escaped Cn cells into new host cells (Figure 1A, yellow arrows and Supplemental Videos 1 and 2), direct cell-to-cell movement (Figure 1B, red arrows) and division of infected host cells (Figure 1A, white arrows and Supplemental Video 2 in yellow frame) was also observed. Although the viability of host cells was not compromised following extrusion, after long-term infection in cell culture (>24 hrs), host cells lysed and intracellular Cn were released (Figure 1A, t = 27∶36∶03). Taken together, our time-lapse microscopy analyses indicated that key aspects of host-Cn cell interactions (i.e., pathogen uptake, replication, cell-to-cell dissemination, extrusion, and host cell lysis) were shared by the *Drosophila* S2 host cell model and mammalian macrophages.

**Figure 2:**
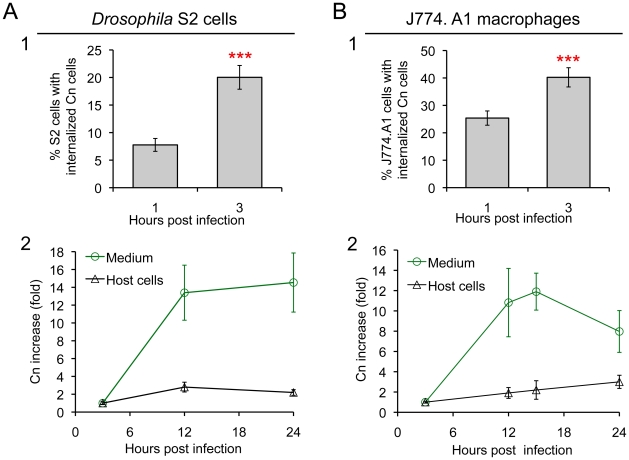
*Drosophila* S2 cells and murine J774.A1 macrophages support similar patterns of Cn phagocytosis and intracellular replication. **A1** and **B1**. Host cell phagocytosis of Cn cells. Infected S2 and J774.A1 cells (on coverslips in 24-well plates) were extensively washed after coincubation with Cn (AI100-dsRed) cells for 1 or 3 hrs. The infected cells were fixed, stained with rhodamine-phalloidin and then analyzed by fluorescence microscopy. The numbers of Cn within host cells were quantified, and the percentages of host cells that had been phagocytosed at the indicated time points post-infection were determined. For each well in each experiment, at least 500 cells were analyzed. **A2** and **B2**. Host cells support Cn intracellular replication and escape from infected cells. At 3 h.p.i., Cn (AI100-dsRed) infected host cells were extensively washed. Fresh medium supplemented with 20 µg/ml fluconazole was added to each well containing infected host cells. The infected cells were then continuously incubated at 28°C (S2 cells) or 37°C, 5% CO_2_ (J774.A1 macrophages). At the indicated time points, media were collected from each well to determine the number of Cn cells that had escaped. The number of CFUs recovered from each sample was then quantified, and the fold-increase of Cn cells (compared to the initial amount recovered at 3 h.p.i.) was calculated and plotted as a function of time. In parallel, at various times post-infection, the corresponding infected S2 and J774.A1 cells were lysed and the number of CFUs contained therein determined after plating and growth on solid medium. The fold-increase of Cn cells was then calculated and plotted (black line) as above. Data represent means ± standard deviation (SD) from at least three independent experiments in which triplicate wells were analyzed.

To determine whether S2 cells constitute an evolutionarily conserved system in which to interrogate Cn interactions with host cells, we employed fluorescence microscopy to visualize eGFP or dsRed-expressing Cn cells (AI132-GFP and AI100-dsRed, respectively) during a time course of infection of S2 or J774.A1 cells. First, we demonstrated that GFP or dsRed expression in Cn cells did not affect pathogen interactions with host cells (Figure S1 A). Next, we employed fluorescence microscopy to analyze the replication of these Cn cells within host cells. For these experiments, we used Alexa 488-conjugated phalloidin or rhodamine-phalloidin to resolve the host cell actin cytoskeleton and thereby demarcate the region occupied by host cells. We then quantified the number of intracellular Cn cells (Figure S1B, upper panel), and found that their number increased over a time course of infection in both S2 and J774.A1 cells (Figure 2, A1 and B1). Interestingly, we also observed Cn cells that appeared to be escaping from S2 cells (Figure S1B, lower panel). These data supported results obtained from our live cell time-lapse microscopy studies (Figure 1 and Supplemental Videos 1 and 2) and encouraged us to develop a system that can differentiate between Cn uptake, replication, and escape in the context of a functional genomic host factor screening experiment.

### Fluconazole protection assay differentiates Cn escape and intracellular replication

Antibiotic protection assays have proven useful for identifying and characterizing interactions between intracellular bacterial pathogens and host cells. Despite the power of these assays, similar systems have not been developed for studying interactions between intracellular fungal pathogens and host cells. We therefore developed such an assay to monitor the intracellular replication and escape of Cn from host cells. Fluconazole is a widely used fungistatic agent that inhibits the growth and proliferation of Cn [49]. To test whether fluconazole differentially affects the intracellular or extracellular populations of Cn cells, we infected *Drosophila* S2 and assorted mammalian cell lines with Cn and then treated the infected cells with various concentrations of fluconazole. We then monitored the growth of the intracellular population by analyzing the number of colony forming units (CFUs) within host cells after lysis and plating onto solid medium. The extracellular populations were also measured by plating media collected from the culture. Finally, the viability of the host cells was assessed in trypan blue exclusion experiments [41]. Fluconazole (0 to 40 µg/ml) had no effect on the proliferation/viability of host cells in culture as demonstrated previously [50] and shown in Figure S2A. However, the replication of Cn cells in fluconazole-treated (20 µg/ml) fresh or conditioned medium (CM) was inhibited (Figure S2B). After 15 hrs, the number of Cn cells decreased in fresh or conditioned mammalian tissue culture medium (Dulbecco's Modified Eagle's Medium: DMEM) at 37°C under 5% CO_2_ (Figure S2B, red line), indicating that prolonged treatment of Cn with fluconazole in DMEM was toxic to Cn cells. However, the number of Cn cells in *Drosophila* fresh or conditioned medium remained at a similar level during 24 hrs of incubation at 28°C in ambient air (Figure S2B, green line). When fluconazole was added to Cn infected S2 or J774.A1 cells, levels of intracellular replication were similar during the first 15 hrs of infection (Figure 2, A2 and B2, black lines).

We were encouraged by these findings and exploited the fluconazole protection approach to evaluate the escape of Cn cells from infected host cells (Figure S2C). When culture media was collected over a 15 hr time period from Cn-infected S2 or J774.A1 cells that had been treated with fluconazole, the number of Cn cells in the media increased dramatically (Figure 2, A2 and B2, green line). The fluconazole containing culture medium did not support the replication of Cn (Figure S2B), which was consistent with the fungistatic nature of this compound. Moreover, fungal cells retained viability in this medium [49]. The dramatic increase of Cn cells in fluconazole-containing culture media could therefore be regarded as derived from a population of Cn cells that had replicated in and escaped from host cells. Therefore, our CFU analysis provided a means of quantifying the number of Cn cells that escaped from host cells over time. The number of Cn cells recovered from the extracellular media of infected J774.A1 cell cultures decreased after 15 hrs (Figure 2, B2, green line), consistent with our observation that prolonged incubation of Cn in fluconazole-containing DMEM impaired Cn viability (Figure S2B, red line). Hence, experiments that exploited fluconazole to measure the amount Cn escape from mammalian cells were not conducted for more than 15 hrs. In contrast, a loss of Cn viability was not observed in the population of escaped cells that were recovered from the supernatants of S2 cell cultures (Figure 2, A2, green line), again consistent with our observation that Cn viability remained at a similar level during 24 hrs of incubation in S2 cell culture supernatants that contained fluconazole (Figure S2B, green line). Taken together, our data indicated that S2 and J774.A1 cells displayed striking similarities in supporting infection by Cn (Figure 1 and Figure 2) and that fluconazole protection assays could be used to assess the intracellular replication and escape of Cn from both host cell types.

### Cn traffics similarly in *Drosophila* S2 and murine J774.A1 cells

We hypothesized that the intracellular trafficking of Cn in S2 and J774.A1 cells share similarities. To test this hypothesis, we used immunofluorescence microscopy assays to compare the intracellular trafficking patterns of AI100-dsRed in these cell types. Antibodies that recognize evolutionarily conserved *Drosophila* and murine proteins that reside in assorted subcellular compartments were used for these experiments (Figure S3A, and [51]). These antibodies were shown to display minimal cross-reactivity with the pathogen in immunofluorescence microscopy experiments (Figure S3B).

**Figure 3:**
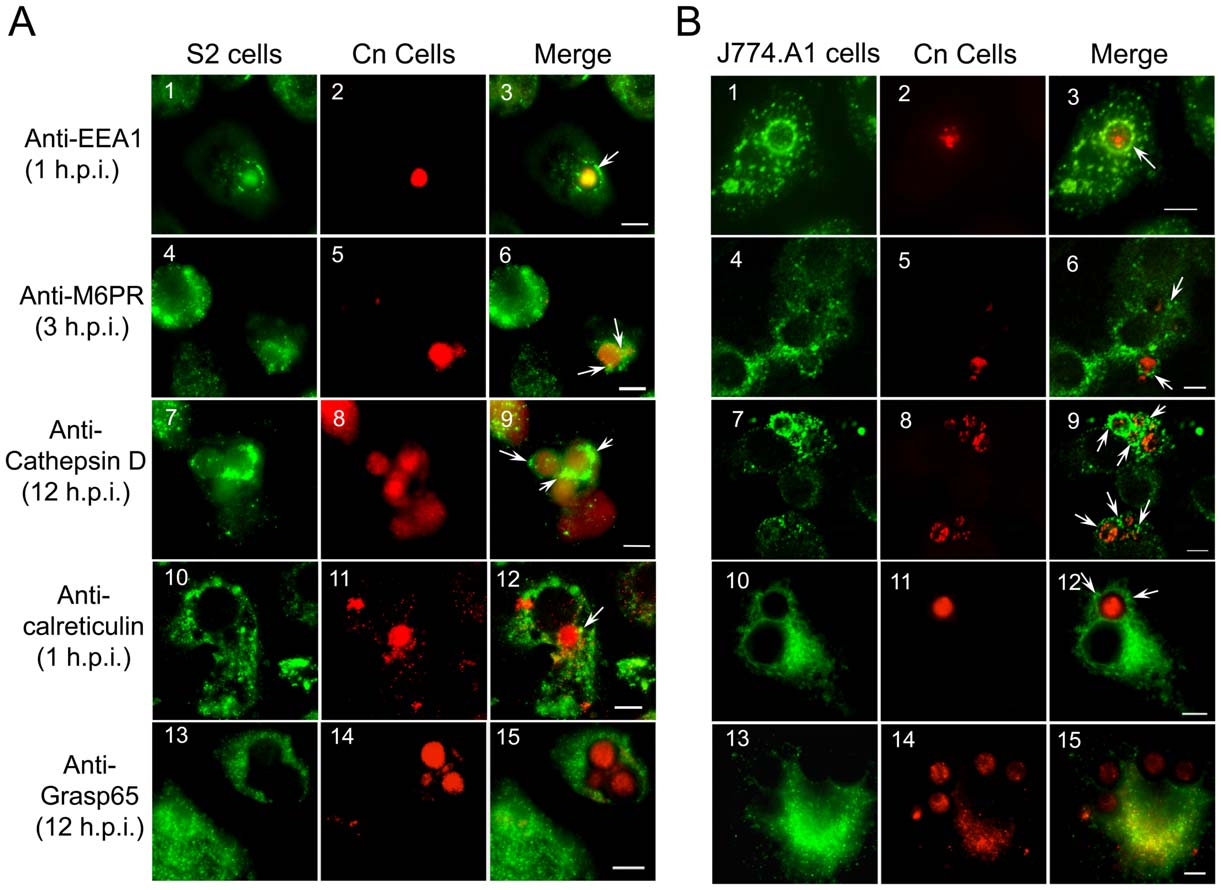
*Cryptococcus neoformans* intracellular trafficking in *Drosophila* S2. (**A**) and J774.A1 (**B**) cells. Host cells were seeded on 12-mm coverslips, placed on the bottom of 24-well plates and then infected with AI100-dsRed. The infected cells were washed three times with 1×PBS at 3 h.p.i., and fresh medium supplemented with 20 µg/ml fluconazole was added to each well containing infected host cells. The infected cells were then continuously incubated at 28°C (S2 cells) or 37°C, 5% CO_2_ (J774.A1 cells). At the indicated time points, the infected cells were fixed and stained for analysis by immunofluorescence microscopy using antibodies directed against the indicated proteins. In Panels **A** and **B**, the localizations of the early endosome marker EEA1 (1), late endosome marker M6PR (4), lysosome marker cathepsin D (7), endoplasmic reticulum (ER) marker calreticulin (10) and Golgi marker Grasp65 (13) in host cells are shown in green. Internalized or replicative Cn cells are shown in red (2, 5, 8, 11, 14). Cn cells in vacuoles that are surrounded by EEA1 (3), decorated with antibodies directed against the late endosome marker M6PR (6) or the lysosome marker cathepsin D (9), or surrounded by calreticulin (12) are shown in the merged images (white arrows). Tight association between Cn and Grasp65 was not observed at the indicated time point (15). Representative images from a single representative experiment are shown. At least three independent experiments were performed and each experiment gave similar results. Scale bars indicate 5 µM.

The movement of Cn from the host cell plasma membrane to its replicative niche involves several sequential steps [16], [19], [20], [21]. Upon entry, Cn cells were localized within a vacuole that contained the early endosome marker EEA1 (Figure 3, A3 and B3). The maturing Cn-containing vacuole (CnCV) could also be sequentially stained with antibodies directed against the late endosome marker mannose-6-phosphate receptor (M6PR), the late endosome/lysosome marker LAMP-1, and the lysosome marker cathepsin D (Figure 3 and data not shown). The pathogen was also observed to be tightly associated with membranes that contained the ER marker calreticulin (Figure 3, A12 and B12). However, Cn interactions with the Golgi marker Grasp65 were not observed at the indicated time point (Figure 3, A15 and B15). Similar findings were obtained in both S2 and mammalian cell lines (Figure 3). Quantitative analysis of these marker protein-positive CnCVs indicated that the host endocytic pathway was involved in the phagocytosis and intracellular trafficking of the pathogen (Figure S4). Therefore, these data indicated the route of intracellular trafficking of Cn in S2 cells, and also demonstrated that Cn intracellular trafficking and replication in S2 and mammalian cells share strikingly similarities.

### Cn wild-type and mutant strains display similar infection profiles in both S2 and mammalian cell systems

**Figure 4:**
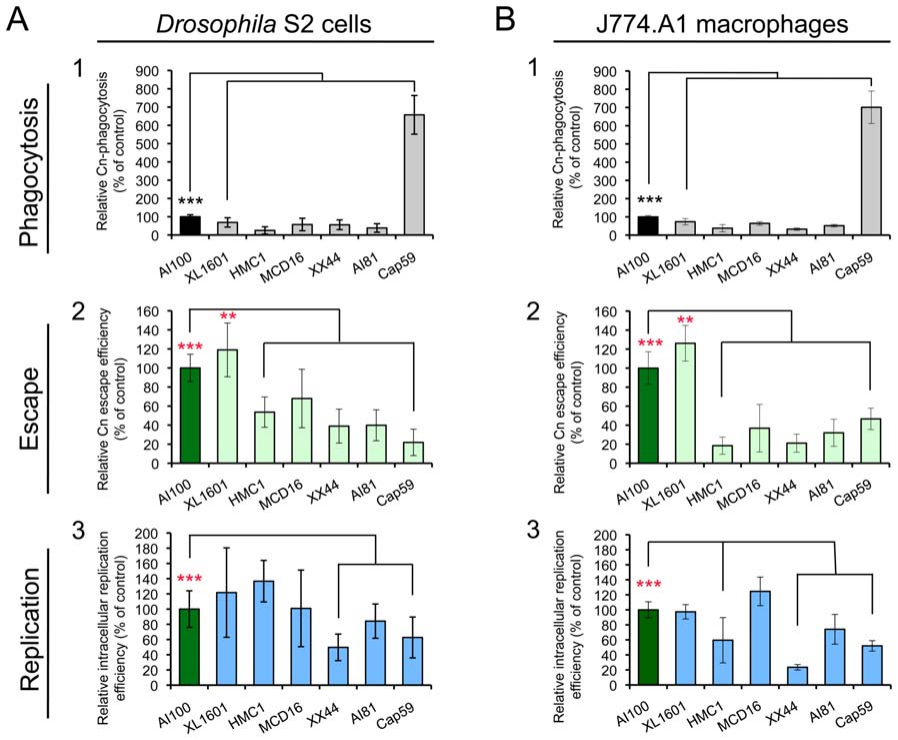
*Cryptococcus neoformans* mutants behave similarly in *Drosophila* S2 cells. (**A**) and murine J774.A1 macrophages (**B**). Host cells were infected with the following Cn strains: AI100-dsRed, XL1601, HMCA1, MCD16, XX44, AI81 and Cap59. The infected cells were then extensively washed at 3 h.p.i. and then fresh medium supplemented with 20 µg/ml fluconazole was added to each well. The infected cells were continuously incubated at 28°C (S2 cells) or at 37°C, 5% CO_2_ (J774.A1 cells). At 3 and 15 h.p.i., the number of Cn cells present in the supernatant or within host cells was determined as described in the *Materials and Methods* section. Cn mutants display similar internalization patterns (**A1** and **B1**), levels of Cn escape (**A2** and **B2**) and relative intracellular replication efficiencies (**A3** and **B3**) in S2 (**A**, left panel) and J774.A1 (**B**, right panel) cells. Data from the control (AI100-dsRed) were normalized to 100%. Data represent the means ± SD from at least three independent experiments. **, *** indicates significance at P<0.01 and P<0.001, respectively.

To further examine whether *Drosophila* S2 cells can serve as a model host cell system for studying host-Cn interactions, the infection phenotypes of several serotypes and mutant variants of Cn (Table S1) were compared in J774.A1 and S2 cells. These mutant strains were selected for study because they were derived from the wild-type (WT) H99, displayed varying degrees of virulence in murine models of cryptococcosis, and possessed defects in distinct genetic pathways (Table S1). When the tested Cn strains were used to infect mammalian macrophages and S2 cells, similar patterns of pathogen uptake, replication and escape were observed (Figure 4 and Figure S5). For example, strain XL1601 displayed similar uptake, replication, and escape efficiencies in both cell systems (Figure 4). The number of XL1601 CFUs that were recovered from inside host cells was similar to that of the AI100-dsRed control (Figure 4, A3 and B3). However, the size of the corresponding extracellular population was larger than the AI100-dsRed control population (Figure 4, A2 and B2), consistent with the possibility that the enhanced capability of this mutant to escape from host cells contributes to its enhanced virulence in animal models of cryptococcosis [52]. The acapsular mutant cap59 displayed ∼6-fold higher rates of phagocytosis than capsular strains (Figure 4, A1 and B1), consistent with previous observations [53]. These results indicated that WT and mutant strains of Cn displayed similar infection profiles in both mammalian and S2 cell systems, thereby supporting the conclusion that the S2 cell system provides a useful model host cell system.

### Cn infection profiles were similar in drug-treated *Drosophila* S2 and J774.A1 cells

If *Drosophila* S2 cells are to provide a model system for elucidating evolutionarily conserved host molecules, then they must share Cn infection phenotypes with mammalian cells when host cell functions are disrupted. With this idea in mind, we examined whether Cn (AI100-dsRed) interacted similarly with S2 and J7774.A1 cells that had been pre-treated (1 hr) and then continuously incubated with a variety of compounds that disrupt host cell functions (Table S2). Fluconazole was added to the media immediately after infection so that the intracellular replication and escape of the pathogen from host cells could be independently monitored using fluconazole protection assays. We found that the tested compounds neither inhibited fungal growth in culture media (Figure S6, A and B) nor compromised the viability/growth of host cells under the examined conditions (data not shown and [41]). We also showed that pretreating (3 hrs) Cn with these compounds at the indicated concentrations before host cell infection did not impair the entry, intracellular replication and escape of the pathogen from host cells (Figure S6C).

**Figure 5:**
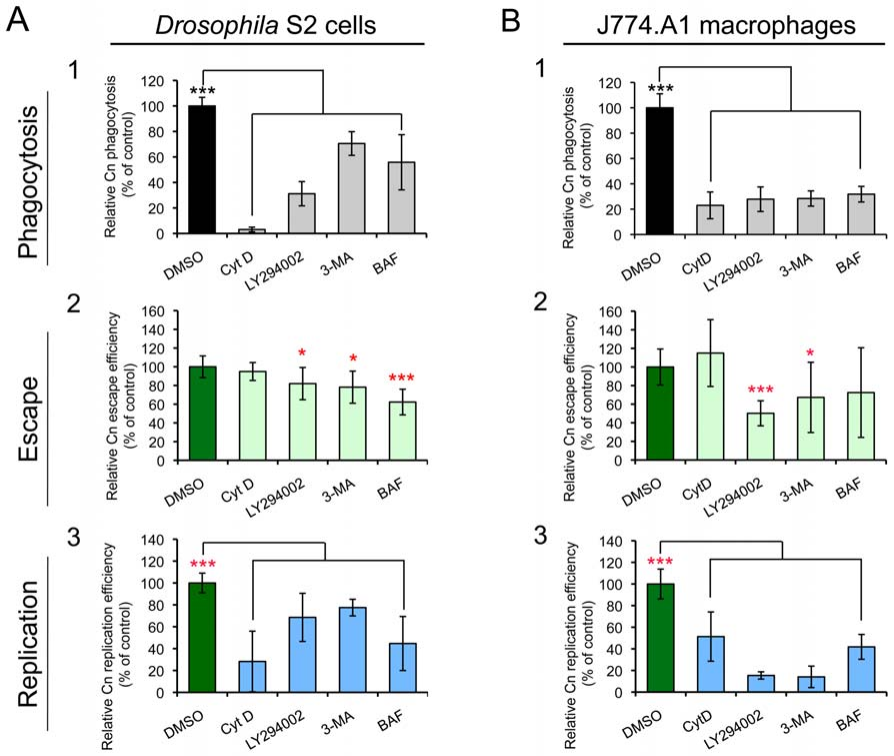
Effect of selected compounds on *Cryptococcus neoformans* infection. *Drosophila* S2 cells (**A**) and murine J774.A1 macrophages (**B**) were coincubated with the indicated compounds for 1 hr at the concentrations shown in Table S2. Next, Cn cells were added to the compound-treated host cells and incubated (in the presence of compound) for 3 hrs. The infected host cells were then extensively washed to remove the extracellular population of Cn cells, and new media containing fresh compounds and fluconazole (20 µg/ml) were added. Phagocytosis of Cn cells by host cells (**A1** and **B1**) was determined at 3 h.p.i. by lysing the infected cells, plating the cell lysate on YPD solid medium, and determining the CFUs present after 24 hr incubation at 37°C. Finally, at 12 h.p.i., the escape (**A2** and **B2**) or intracellular replication (**A3** and **B3**) efficiencies of Cn cells were measured separately as described in the *Materials and Methods* section. *, **, *** indicates significance at P<0.05, 0.01 and 0.001, respectively, compared with the DMSO treated control. CytD, 3-MA and BAF indicate that host cells were treated with cytochalasin D, 3-Methyladenine and bafilomycin A1, respectively. Data represent the means ± standard deviations from at least three independent experiments.

We examined whether compound-treated S2 and J774.A1 cells supported similar patterns of pathogen uptake and replication. Treatment of either cell type with cytochalasin D (CytD), which disrupts actin cytoskeleton polymerization, dramatically reduced the amount of Cn phagocytosis. However, this compound did not inhibit the escape of replicative Cn cells from host cells (Figure 5, A2 and B2). When host cells were treated with LY294002 or 3-Methyladenine (3-MA), which disrupts class III PI3-kinase activity [54], [55], the entry, intracellular replication and escape of Cn from host cells were significantly reduced (Figure 5). Finally, bafilomycin A1 (BAF), an autophagy inhibitor that disrupts vacuolar H^+^-ATPase activity and endolysosomal acidification [56], significantly inhibited intracellular replication of internalized Cn cells. However, this compound had limited effect on the escape of Cn cells from mammalian macrophages (Figure 5). These data indicated that the infection profiles of Cn were similar in *Drosophila* S2 and J774.A1 cells that had been treated with compounds that disrupt host cell functions.

### RNAi screen identifies host factors that mediate Cn infection

Encouraged by our findings that Cn infection of S2 cells recapitulates salient aspects of the corresponding infection of mammalian cells, we examined whether the S2 cell system and RNAi technology could be combined to identify host factors that mediate host-Cn interactions. We used a novel RNAi screening approach (Figure S7) to perform a pilot screen for these host factors. Cn is known to replicate within a poorly characterized phagosome-derived compartment [21], [22], [57], suggesting that host factors involved in membrane remodeling, trafficking, or phagosome biogenesis and maturation may be critical to the intracellular lifestyle of the pathogen. For our screen, we pre-selected 410 dsRNAs that targeted the knockdown of genes that (1) had been annotated to be associated with these events (http://flybase.org/) and (2) were available in Release 1.0 of the *Drosophila* RNAi Library (Open Biosystems, Huntsville, AL, USA). After two rounds of screening, sixty-two dsRNAs that significantly altered Cn infection of S2 cells were identified. Among the 62 candidates, 57 high priority hits (Table S3) were identified after three rounds of screening. A hit was defined as a dsRNA treatment in which the relative infection index (RIF, see the *Materials and Methods*) differed by two fold of the standard deviation (SD) from the untreated control. We also screened 96 dsRNAs that were randomly picked from one of the 76 source plates in the *Drosophila* RNAi library. Because the manufacturer randomly arrayed dsRNAs into source plates, this strategy for selecting dsRNAs introduced no bias in gene function into this analysis. Six of the randomly selected dsRNAs impaired host cell growth. However, after two rounds of screening, dsRNA hits were not uncovered in this experiment. Therefore, we estimate the hit frequency in the whole *Drosophila* genome to be less than 1%, and note that our preselected set of dsRNAs was significantly enriched in dsRNAs that target Cn host cell functions.

**Figure 6:**
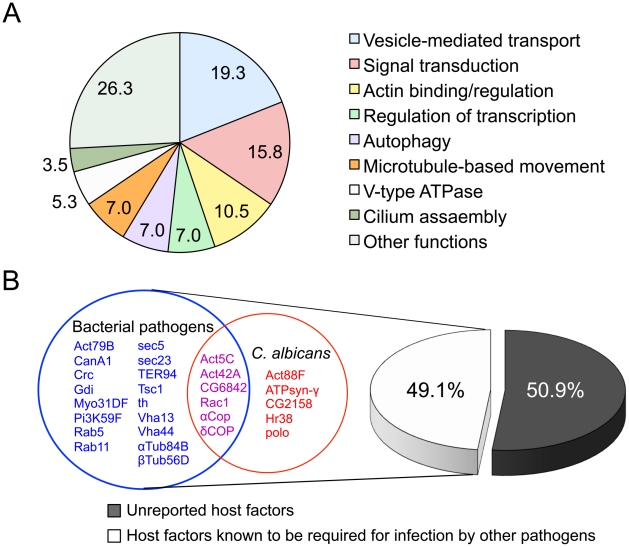
Identification of host factors regulating *Cryptococcus neoformans* infection. **A**. Fifty-seven host factors that, when depleted, significantly altered Cn infection phenotypes were identified in an RNAi screen using *Drosophila* S2 host cells. The 57 host factors were analyzed using established gene ontology categories (http://flybase.org/). The results were plotted in a pie chart with the percentage of genes in each category indicated. **B**. Comparison of the 57 identified host factors with those that were reported to mediate bacterial or fungal pathogen infection of host cells. Among the 57 hits, twenty-nine (50.9%) had not been previously reported to function as host factors for intracellular fungal pathogens. Shared and unique host factors were determined by analyzing published data from screens performed in S2 cells infected with the following pathogens: *Brucella melitensis* [41], *Chlamydia caviae* [43], *Chlamydia trachomatis* [44], *Listeria monocytogenes* [38], [40], *Mycobacterium fortuitum* [40], [42] and *Candida albicans* [39]. The host factors that are shared with other human pathogens (49.1%) are listed in red (*C. albicans*), magenta (*C. albicans* and bacterial pathogens) and blue (bacterial pathogens). Each host factor mediates Cn phagocytosis and/or intracellular replication.

We analyzed our high priority hits in several ways. First, we classified hits into functional classes, including vesicle-mediated transport proteins, cytoskeletal proteins, and vacuolar proton-transport factors (Figure 6A and Table S3), according to the gene ontology system of biological and molecular function, cellular component, or protein domains as reported in FlyBase (www.flybase.org). Interestingly, of the 57 high priority hits, twenty-eight hits (49.1% of the total hits recovered) had been previously shown to mediate infection of S2 cells by fungal or intracellular bacterial pathogens, including *Brucella melitensis* [41], *Chlamydia caviae* [43], *Chlamydia trachomatis* [44], *Listeria monocytogenes* [38], [40] or *Mycobacterium fortuitum* [40], [42] (Figure 6B and Table S3). Among the 28 hits, 11 genes (19.3% of the hits) are known to be required for phagocytosis of *C. albicans* [39], a fungal pathogen that causes oral and genital infections in humans and is also associated with morbidity and mortality in immunocompromised patients (Figure 6B and Table S3). Several host factors previously known to function in phagocytosis [39], such as Rac1, actins, and PI3-kinase (class III) and COPI coatomers, were identified as high priority hits (Table S3). Consistent with these findings, pharmacological disruption of the functions of these host factors with CytD or LY294002 reduced host cell phagocytosis of Cn cells (Figure 5, A1 and B1). Finally, we identified 29 genes (50.9% of the high priority hits) that had not been previously reported to be involved in mediating intracellular Cn infection (Table S3). Therefore, our screening strategy was sufficiently robust to uncover suspected host factors as well as factors with novel functions.

### A role for host cell autophagy proteins in mediating Cn infection

**Figure 7:**
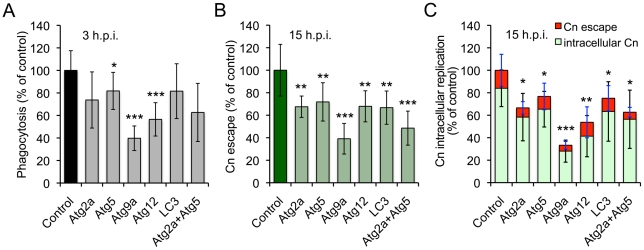
Host Atg proteins mediate *Cryptococcus neoformans* infection of host cells. Murine RAW264.7 macrophages were transfected with the indicated siRNAs (10 nM). Two days post-transfection, the host cells were infected with Cn (H99) for 3 hrs. The infected cells were extensively washed to remove the extracellular population of Cn cells, and fresh medium supplemented with 20 µg/ml fluconazole was added. At the indicated time points post infection, the supernatant and lysate from infected cells were collected separately. The materials were then plated, and the number of CFUs per well was determined. The resultant CFUs were used to calculate the relative phagocytosis (**A**), Cn escape (**B**) and intracellular replication (**C**) as described in the *Materials and Methods*. Data represent the means ± SD from at least four independent experiments with triplicate wells examined for each treatment. *, **, *** indicates significance at p<0.05, 0.01 and 0.001, respectively.

Our screen uncovered several autophagy (Atg) genes, including *Atg2* (CG1241), *Atg5* (CG1642) and *Atg9* (CG3615), which play conserved roles in autophagosome biogenesis [58]. However, the engagement of host cell Atg proteins by Cn had not been previously reported. To determine whether elements of the host cell autophagy pathway regulate Cn interactions with macrophages, we exploited murine RAW264.7 macrophages as a host cell system. RAW264.7 cells provided an established model for interrogating Cn interactions with mammalian macrophages [9]. RNAi mediated gene knockdown can also be readily achieved in these cells, which was advantageous for our studies. We thus treated RAW264.7 cells with siRNAs to knock down the expression of the murine orthologs of several *Drosophila* Atg genes that were uncovered in our S2 cell screen. The targeted mammalian genes included *Atg2a*, *Atg5*, *Atg9a*, *Atg12* and *LC3*. After validating gene knockdown by immunoblotting analysis (Figure S8), we infected the siRNA treated cells with Cn (H99), and then quantified the phagocytosis, replication, and escape of the pathogen using our fluconazole protection assay. When host cells that had been depleted of the indicated Atg proteins were infected with Cn, the uptake and/or replication of the pathogen were significantly decreased compared to controls that had been treated with scrambled siRNAs (Figure 7). These data supported findings obtained from our RNAi studies using the S2 cell system (Table S3) that indicated the engagement of host cell autophagy proteins in supporting Cn infection.

### Host Atg proteins are recruited to Cn-containing vacuoles during infection

**Figure 8:**
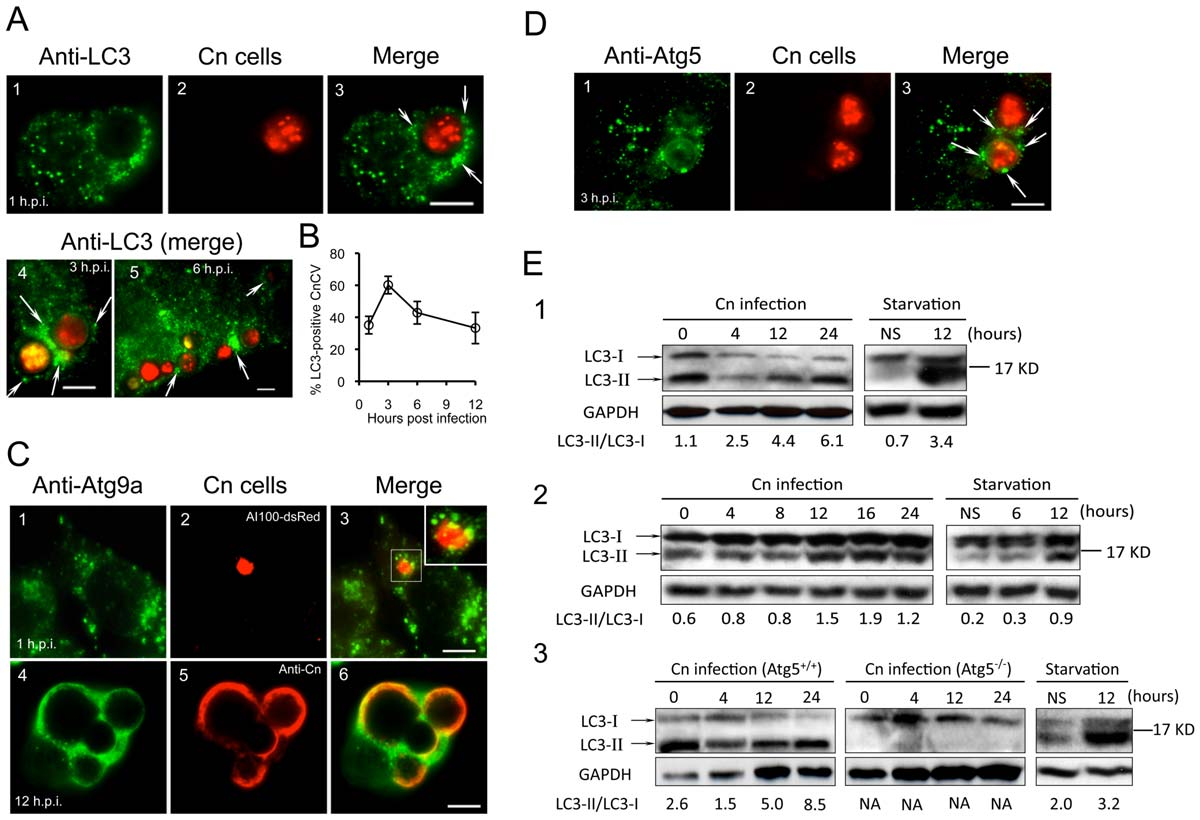
*Cryptococcus neoformans* interactions with Atg proteins during infection. Murine J774.A1 or RAW264.7 cells were seeded on 12-mm coverslips placed on the bottom of 24-well plates and then infected with Cn (AI100-dsRed) for 1 or 3 hr. At 3 h.p.i., the infected cells were wash three times and then continuously incubated in fresh medium supplemented with 20 µg/ml fluconazole. At the indicated time points, the infected cells were fixed and prepared for immunofluorescence microscopy using antibodies directed against mouse proteins LC3 (**A**, J774.A1 cells) or Atg9a (**C**, RAW264.7 cells. Inset in **C3**: a magnified section shows Atg9a in close apposition to CnCvs during an early stage of infection) and Atg5 (**D**, J774.A1 cells). Regions of colocalization appear yellow in the images (**C3** and **C6**). CnCVs surrounded by host proteins are indicated by white arrows. Scale bars indicate 5 µM. Images were taken from a representative experiment of at least three independent experiments. **B**. Quantification of Cn cells that resided in host LC3-positive compartments during a time course of infection. At each time point, the percent of total cells analyzed in which LC3 was tightly associated with CnCvs was calculated and plotted as a function of time. Data represent the means ± SD from at least three independent experiments. For each experiment, at least 300 internalized or replicative Cn cells were analyzed. **E**. Induction of LC3-I to LC3-II processing by Cn infection. Murine J774.A1 (**E1**), RAW264.7 (**E2**) macrophages or MEFs (**E3**) were infected with Cn strain AI100-dsRed or cultured in serum free DMEM (starvation) for 12 hr. At the indicated time points, Cn infected and serum starved cell samples were collected, and subjected to immunoblotting analysis using antibodies directed against mouse LC3. NS = non-starved. Representative images were taken from a single experiment. Three independent experiments gave similar results.

Our siRNA experiments in RAW264.7 cells suggested that Atg2, Atg5 and Atg9 support Cn infection of both *Drosophila* S2 and mammalian cell systems. To further investigate the roles of Atg proteins in supporting pathogen infection, we employed confocal immunofluorescence microscopy to analyze the interaction of assorted Atg proteins with Cn cells during a time course of Cn infection of mammalian host cells. Upon internalization, Cn was observed in close apposition to membranes that were decorated with LC3, Atg9a and Atg5 (Figure 8, A–D), suggesting that these autophagy elements are recruited to the vicinity of CnCVs. Interestingly, the intimate interactions between Cn and these Atg proteins during the early stages of Cn infection were observed in several mammalian host cell types, including J774.A1, MEF and RAW264.7 cells (Figure 8, A–D and data not shown). Quantitative analysis revealed that the recruitment of Atg proteins to the vicinity of CnCVs was reduced after 3 h.p.i. (Figure 8, A and B, white arrows and data not shown). These microscopy experiments therefore supported findings from our siRNA experiments.

### LC3-I conversion to LC3-II during Cn infection

To further investigate the possibility that the activation of proteins in the autophagosome biogenesis pathway supports Cn infection, we examined the conversion of host LC3-I to LC3-II as an indicator of these events. As expected, overnight nutritional limitation of host cells induced the conversion of LC3-I to LC3-II, resulting in an increased ratio of LC3-II/LC3-I (Figure 8E); however, this conversion was not significantly increased over a 24 hr time course of incubation in the absence of Cn infection (Figure 8E and data not shown). Immunoblotting results indicated that the level of LC3-II, and the ratio of LC3-II/LC3-I increased during Cn infection of WT J774.A1, RAW264.7 and Atg5^+/+^ MEF cells (Figure 8E). However, the conversion of LC3-I to LC3-II was not detected in Atg5^−/−^ MEFs at all of the tested time points (Figure 8E3). This latter finding was consistent with our observations that Cn cells did not reside in LC3-positive phagosomes/vacuoles in the Atg5^−/−^ MEFs (data not shown). Taken together, our data demonstrated that Cn infection of host cells involves interactions with LC3-positive phagosome membranes.

## Discussion

Yeast or basidiospore forms of Cn typically use the host respiratory system to gain entry into the host. Cn then rapidly disseminates to extrapulmonary tissues, including the brain, which accounts for the meningoencephalitis that is typically associated with cryptococcosis. Macrophages constitute important cellular targets of infection. Cn infects and replicates within alveolar macrophages [17], [59], and can also exploit macrophages to gain entry into the central nervous system via a Trojan horse mechanism in which these immune cells ferry ingested Cn cells across the blood brain barrier [60], [61]. Understanding host mechanisms that support the survival and replication of the pathogen within macrophages is therefore important to understanding pathogen dissemination and disease progression.

Non-vertebrate models have emerged as tractable experimental systems for illuminating host-pathogen interactions [62], [63]. The current study indicates that the combination of macrophage-like *Drosophila* S2 cells and RNAi technology provide an appealing system for elucidating the roles of host factors in mediating the host-Cn interaction. Cn infection of S2 cells recapitulated salient aspects of mammalian macrophage cell infection. First, Cn infection of S2 and mammalian cells shared similar infection phenomena, including phagocytosis, intracellular replication, cell-to-cell dissemination, extrusion and host cell lysis profiles. Moreover, the intracellular trafficking and replication of Cn in murine macrophages and *Drosophila* S2 cells shared striking similarities, suggesting that the fungal pathogen subverts conserved host factors to secure an intracellular replicative niche and to disseminate. Second, Cn strains from different genetic backgrounds and mutants with altered virulence in mouse models of infection displayed similar patterns of uptake and replication in S2 and J774.A1 cells. Third, similar patterns of Cn phagocytosis, intracellular replication, and escape were observed in host cells that had been treated with structurally diverse compounds that target specific host cell functions. These data suggested that both host cell systems share common molecular requirements to support these processes. Fourth, results from an RNAi screening experiment demonstrated that host factors previously known to be required for the phagocytosis of various bacterial and fungal pathogens [39] are also required for Cn infection of host cells (Table S3 and Figure 6B). These data support the idea that evolutionarily conserved host functions support pathogen interactions with S2 and mammalian cells. Finally, Cn cells have been isolated from insects such as cockroaches and beetles [45], [46], which is consistent with the possibility that aspects of the observed interactions with S2 cells may recapitulate pathogen interactions with insects in the natural environment. It has been postulated that the pathogenic strategies of Cn in mammals evolved from interactions between Cn and its natural environmental predators [64]. Our data support the conclusion that S2 cells provide a useful model system for elucidating host-Cn interactions.

Autophagy is a catabolic process in which cytosolic constituents are sequestered from the rest of the cytoplasm by the autophagosome [27], which delivers the sequestered constituents to lysosomes for degradation. Autophagy is a tightly regulated process that is essential for survival, differentiation, development and homeostasis [25], [26]. Several forms of autophagy have been identified that differ with respect to their physiological functions and the mode of cargo delivery to the lysosome [25]. Atg5 is essential for most forms of mammalian autophagy [65], [66], [67]. This protein mediates the conversion of LC3-I to LC3-II, which drives autophagosome biogenesis, and indicates activation of the autophagy pathway. Atg5 also directs the localization of LC3-II to autophagosomes [28], [29]. Atg9a, the only transmembrane Atg protein in mammals, is proposed to mediate membrane transport to generate autophagosomes [68]. The Atg18-Atg2 complex is involved in the transport of Atg9 to autophagic membranes [69], [70]. Finally, an alternative, Atg5/Atg7-independent macroautophagy pathway has been described in which conversion of LC3-I to LC3-II does not occur [71], [72]. Therefore, autophagy can be engaged without recruiting the full complement of Atg proteins that have been annotated to be associated with this process.

Recent studies indicate that autophagy may be an important pathogen control mechanism [24], [25], [26]. Atg5 plays roles in inhibiting the growth of pathogens, such as *L. monocytogenes* [30], [31], [32] and *T. gondii* [32], [33]. In *Drosophila*, autophagy helps control *L. monocytogenes* infection of primary hemocytes [31]. In contrast to the potential protective role for autophagy against bacterial and parasitic infection, other studies suggest that several bacteria and parasites may subvert host autophagic processes to establish a successful infection; these pathogens include *Staphylococcus aureus* [73], *Legionella pneumophila* [74], *B. melitensis* [41], *C. burnetii* [75], *Helicobacter pylori* [76], [77], *Trypanosoma cruzi* [78] and *T. gondii* [79]. In addition, some pathogens, such as the HSV-1 virus, are known to exploit elegant mechanisms for inhibiting signaling processes that induce autophagy [80], [81]. *T. gondii* infection induces host cell autophagy *via* a mechanism that depends on host Atg5. In fact, the growth of this parasite is defective in Atg5-deficent cells [79]. Therefore, in addition to their roles in the canonical autophagy pathway, host Atg proteins may also contribute to modulating the level of replication of viral or intracellular pathogens.

In this study, we demonstrated that Cn exploits host cell autophagy proteins to secure an intracellular replicative niche. First, we showed that replicative Cn cells reside in CnCVs that are tightly associated with the autophagosome marker LC3 and lysosomal marker LC3. However, similar LC3 localization was not observed in pathogen-infected Atg5^−/−^ MEFs. Second, we demonstrated that compounds that are known to disrupt the activities of proteins that regulate the host autophagy pathway (BAF, LY294002 and 3-MA) inhibit Cn uptake and replication. In an early stage of autophagosome formation, the Beclin 1-hVps34 (class III PI3-kinase) complex supplies phosphatidylinositol-3-phosphate (PI[3]P) to the pre-autophagosome membrane, a process that is essential for autophagosome membrane formation [82]. 3-MA and LY294002 are inhibitors of class III PI3-kinase and also block autophagy [54], [55]. Our data demonstrate that host cells treated with 3-MA or LY294002 display reduced levels of Cn infection, and implicate PI3-kinase activity in supporting the intracellular lifestyle of the pathogen. Finally, we showed that RNAi-mediated depletion of host Atg proteins, including Atg2a, Atg5, Atg9a, reduced Cn infection of host cells. RNAi-mediated depletion of Pi3K59F (class III PI3-kinase) also failed to support Cn infection in S2 cells. Taken together, our data demonstrate that Cn exploits host proteins that regulate the host autophagy machinery to facilitate intracellular replication. Interestingly, Cn employs autophagy as a survival mechanism and virulence-associated trait. Cn strains lacking the Vps34 PI3-kinase and (vps34Delta) and depletion of Atg8 markedly attenuated virulence in a mouse model of infection [83]. Therefore, autophagy pathways in both the host and pathogen play important roles in supporting the interaction between Cn and host cells.

**Figure 9:**
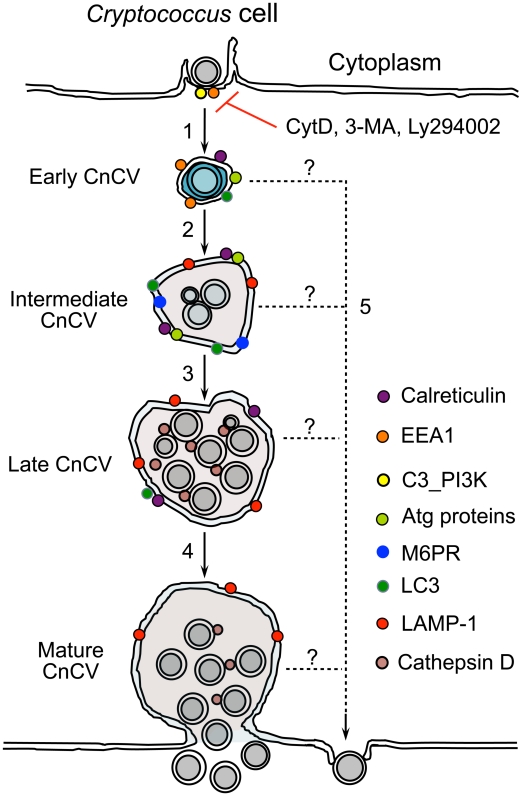
Proposed model depicting Cn subversion of Atg proteins for intracellular replication and dissemination. Host cell phagocytosis of Cn cells is class III PI3-kinase (C3_PI3K) and actin-dependent. This process is blocked by the PI3-kinase inhibitors 3-MA, LY294002 and the actin polymerization inhibitor cytochalasin D (CytD) (1). Internalized Cn resides in a Cn containing vacuole (CnCv). Early CnCvs interact with components of the endocytic pathway and sequentially obtain early endosome (EEA1), late endosome (M6PR) and lysosome (LAMP-1 and cathepsin D) markers (2). Infection by Cn may also activate the host cell autophagy pathway. Atg proteins such as Atg5, Atg9a and LC3 are recruited to the vicinity of CnCVs. Activated host cell Atg proteins promote the maturation of early CnCVs to form intermediate CnCVs (2). These compartments support Cn replication (3). Finally, late CnCVs may fuse with the plasma membrane, via an undefined mechanism, which results in the escape of replicative Cn cells from infected host cells (4). Extrusion in the absence of Cn replication and/or CnCv maturation may also occur (5).

Based on our findings, we propose a model in which key elements of the autophagy pathway, including Atg5-Atg12, Atg9a and LC3, coordinate with other host factors identified in our RNAi screen to regulate the sequential intracellular replication and escape of Cn from host cells (Figure 9). The following step-wise events are envisioned. First, phagocytosis of Cn is mediated by several factors, including host actins and class III PI3-kinase activities. Upon entry into host cells, Cn traffics to EEA1-positive CnCVs, which then traffic along the endocytic pathway, interact with membranes containing late endosome markers and autophagosomal markers (LC3), and fuse with lysosomes to become late CnCVs that are decorated with markers associated with endosome/lysosome (LAMP-1) and lysosome (cathepsin D) proteins. These compartments are permissive for Cn intracellular replication. Finally, Cn cells can escape from CnCvs at any stage of the maturation process (Figure 9).

Intracellular and extracellular populations of Cn each contribute to disease progression in the infected host. For example, enlarged Cn cells that resist phagocytosis have been reported [84], [85], [86], [87]. Strains that harbor mutations in signaling pathways of the pathogen that support giant cell formation display reduced virulence [85]. These observations are consistent with the conclusion that this extracellular population of Cn cells is important for the infection and dissemination of the pathogen. On the other hand, several lines of evidence support the conclusion that macrophage invasion and replication constitutes an important to disease progression in humans and whole animal model systems. For example, animals depleted of macrophages by clodronate treatment display reduced pathogen load in a variety of organs after infection with Cn [88]. Moreover, *Cryptococcus gatti* strains with enhanced abilities for intracellular replication display enhanced virulence in humans and murine models of infection [89]. The proposed model of intracellular trafficking dynamics put forth in this report will thus illuminate an important aspect of fungal pathogenesis.

## Materials and Methods

### *Cryptococcus neoformans* strains

The *C. neoformans* strains used in this study are listed in Table S1. Yeast forms of Cn cells were grown on YPD (Difco) agar plates and maintained on the plates for no more than one week prior to experimentation. For infection, 2.4 ml of YPD broth was inoculated with a loop of Cn cells taken from a freshly streaked YPD plate. Cultures were then grown overnight with shaking at 37°C.

### Cell culture

*Drosophila* S2 cells were maintained at 25°C in Insectagro DS2 serum free medium (DS2-SFM, Cellagro) with or without 10% fetal bovine serum (FBS). Murine J774.A1 and RAW264.7 macrophages and Atg5-deficient (Atg5^−/−^) MEFs and the corresponding control (Atg5^+/+^) [65] were routinely incubated at 37°C in a 5% CO_2_ atmosphere in DMEM supplemented with 10% FBS. Cells were seeded in 24 or 48-well plates and cultured overnight before infection. For CFU assays, 1.0×10^6^ S2 and 2.5×10^5^ mammalian cells were seeded in each well of a 24-well plate; for a 48-well plate, 5.0×10^5^ S2 and 1.2×10^5^ mammalian cells were seeded; for fluorescence microscopy assays, 6.0×10^5^ S2 and 1.0×10^5^ mammalian cells were seeded on 12-mm glass coverslips (Fisherbrand) that were placed on the bottom of 24-well plates. The seeded S2 and mammalian cells were cultured overnight before infection.

### *Cryptococcus neoformans* infection

*rosophila* S2 and mammalian cells were infected with Cn cells at a multiplicity of infection (MOI) of 5 and 10, respectively, unless otherwise indicated. Infected cells were then incubated at 25°C (S2 cells) or 37°C in a 5% CO_2_ atmosphere (mammalian cells). One or 3 h.p.i., culture media was removed, and the infected cells were extensively (6 to 8 times) washed with 1×phosphate buffered saline (PBS) (S2 and J774.A1cells) or a mixture of 1×PBS and DMEM with 10% FBS (V∶V = 1∶1) (RAW264.7 cells and MEFs). Fresh media, supplemented with fluconazole (20 µg/ml), was added to each well containing infected host cells and the infected cells were continuously incubated in this antifungal agent for various lengths of time at the indicated conditions. At the indicated time points, culture medium (containing escaped Cn cells) and the corresponding Cn infected host cells were collected separately to measure the number of CFUs. Fluconazole inhibition of Cn replication in S2 and mammalian cell media was also measured by inoculating Cn in fresh or conditioned medium containing 20 µg/ml fluconazole and performing CFU assays. Cn intracellular replication or escape efficiencies for experiments (in which infection was initiated at starting time point t_0_ and proceeded to time point t_n_) were calculated as described in the *Mathematical Formulae* section (see below). If no CFUs were recovered from the culture medium at t_0_, then we could not calculate escape efficiency. In this case, the net increases in the number of CFUs recovered from the media for the experimental treatments were compared to the corresponding net increase of the control at t_n_.

### *Cryptococcus neoformans* CFU/fluconazole protection assays

We used fluconazole protection assays to measure the number of intracellular Cn and also the number of Cn that had escaped from these same host cells. To measure the intracellular population of Cn cells, we infected host cells for 3 hrs, washed these cells extensively (6–8 times), and then added fresh medium containing fluconazole (20 µg/ml). The cells were then continuously incubated for various lengths of time. At the indicated time points, the media was removed. The cells were then lysed by the addition of 0.5% Tween-20 in sterile water (100 µl) and incubation at room temperature (S2 cells) or at 37°C (mammalian cells) for ∼10 min. Additional 100 µl of YPD was added to the resultant whole cell lysates (which contained the intracellular Cn population). Finally, 5 µl of cell lysates were plated onto solid YPD medium after serial dilution. CFUs were counted after 24 hrs of incubation at ∼30°C. To measure the escaped Cn cells, the removed media were transferred to 96-well plates (if total volume <300 µl) or 1.5 ml microcentrifuge tubes (if total volume >300 µl) and centrifuged at 1200×g for 12 min. The media with fluconazole were then removed and the Cn cells were re-suspended in YPD broth. The Cn suspension was serially diluted in YPD broth or 1×PBS buffer, and 5 µl of the diluted Cn suspension was plated onto solid YPD agar plates to determine CFUs as above. To measure internalized Cn cells, after extensive wash, the infected cells were lysed and performed CFU assay as described above. To measure the number of Cn cells in the culture media at the starting time point (t_0_, 3 h.p.i.), after extensive wash (6∼8 times), fresh media (200 µl) without fluconazole or 1×PBS were added to the wells containing infected host cells. The supernatants (which contained the extracellular Cn population) were transferred to 96-well plates immediately after the plates containing infected cells were briefly shaken. After serial dilution, the supernatants were then performed CFU assay as described above.

### Viability of host cells

Host cells were coincubated with or without pharmacological compounds at the indicated concentration (Table S2) for various lengths of time. Quantification of the viability of treated S2 and mammalian cells as well as the total cell number was performed as previously described [41].

### Fluorescence Microscopy and Immunofluorescence Microscopy Assay (IFMA)

To visualize intracellular populations of Cn cells, host cells (e.g., S2 or J774.A1 cells) were seeded on 12-mm coverslips that had been placed on the bottom of wells of 24-well plates. Next, these cells were infected with Cn strain AI100-dsRed or AI132-GFP. At 1 or 3 h.p.i., the infected host cells were extensively washed and then continuously incubated in fresh medium supplemented with 20 µg/ml fluconazole. At different time points post infection, the coverslips with infected host cells were fixed and stained with Alexa 488-conjugated phalloidin (for AI100-dsRed infected cells) or rhodamine phalloidin (for AI132-GFP infected cells) to resolve the host cell actin cytoskeleton. The infected cells were then visualized by confocal fluorescence microscopy. Differential interference contrast (DIC) and fluorescence images were used to analyze phagocytosis of Cn cells by host cells. To elucidate *Cryptococcus* intracellular trafficking and interactions with host proteins, *Drosophila* S2, J774.A1 and RAW264.7 cells that were seeded onto 12-mm coverslips on the bottom of 24-well plates were infected with AI100-dsRed for various lengths of time. To analyze infections of less than 3 hrs, the infected cells were washed three times with 1×PBS or the mixture of PBS and DMEM with 10% FBS, and then the infected cells were fixed before IFMA. Otherwise, at 3 h.p.i., the infected cells were washed three times with 1×PBS or a mixture of PBS and DMEM. In experiments in which the cross-reactivity of extracellular Cn cells with the indicated antibodies was assessed, the Cn infected host cells were washed only once. Next, fresh media supplemented with 20 µg/ml fluconazole were added to the wells containing Cn infected host cells. At various time points post infection, the infected cells were fixed and processed for IFMA as previously described [41] with the following modification: before incubation with antibody, the infected cells were blocked with 10% non-fat dry milk in PBSTT buffer (0.05% Triton X 100+0.05% Tween 20 in 1×PBS) for ∼2 hrs at room temperature, and then the cell samples were incubated with antibody in PBSTT with 5% non-fat dry milk. The primary antibodies used were as follows: goat-anti-human EEA1; rabbit anti-human M6PR; rabbit anti-human LAMP-1; rabbit anti-human cathepsin D; rabbit anti-mouse LC3; rabbit anti-Calreticulin; purified Grasp65 from rabbit; mouse anti-human Atg5; mouse anti-Cn (Meridian Life Science, Inc., USA). Samples were stained with Alexa Fluor 488-conjugated and/or Alexa Fluor 594-conjugated secondary antibody (Molecular Probes, 1∶1000). Acquisition of confocal images, image processing and analyses were performed as previously described [41].

### Live cell imaging

J774.A1 or *Drosophila* S2 cells were seeded in 6- or 24-well plate with glass bottom. The cells were overnight cultured before Cn (AI100-dsRed) infection (MOI = 3). At 3 h.p.i., Cn infected host were washed 3 times with 1×PBS. Fresh media with 20 µg/ml fluconazole were added to the infected cells, and the cells were then incubated in the small environmental chamber (28°C for S2 cells and 37°C, 5% CO_2_ for J774.A1 macrophages) mounted on the laser confocal microscopy (Eclipse Ti, Nikon). Live cell images were automatically taken every 2 min for 24 (J774.A1) and 48 (S2 cells) hrs with NIS elements AR 3.0 software (Nikon), which was initiated after phagocytosing. Images were processed with NIS elements AR 3.0 software.

### Drug treatments

In 48 well plates, *Drosophila* S2 and murine macrophage J774.A1 cells were coincubated with assorted pharmacological compounds including CytD, LY294002, 3- 3-MA and BAF at the indicated concentrations (Table S2). Cells were treated with these drugs 1 hr before and during infection with Cn strain AI100-dsRed. The treated cells were incubated at 25°C (S2 cells) or at 37°C with 5% CO_2_ (J774.A1 macrophages). At 3 h.p.i., the cells were extensively washed with 1×PBS. Fresh media supplemented with the same concentration of the drugs and 20 µg/ml fluconazole were then added to the infected cells. Fluconazole was used to inhibit the replication of extracellular Cn cells, if any. To evaluate phagocytosis of Cn cells, the infected cells (3 h.p.i.) were lysed with 0.5% Tween 20 after extensive washing with 1×PBS. The cell lysate was used to perform CFU assays as described above. In addition, the media were separately collected for CFU assays which determined the initial number of Cn cells present in the media at the starting time point (t_0_). At 12 h.p.i., the media and extracts derived from infected cells were also analyzed using CFU assays as described above. To evaluate the effects of drugs on Cn pathogenicity, the Cn cells were pre-treated with the indicated drugs for 3 hrs and then the drugs were washed out. These cells were then used to infect normal host cells. Phagocytosis, intracellular replication and escape of the drug pre-treated Cn cells were analyzed as described above.

### Generation of dsRNAs

Generation of dsRNAs that target the knockdown in the expression of *Drosophila* proteins were performed as previously described [41]. The dsRNA products were stored at −80°C until use.

### RNAi screen for host factors mediating Cn infection

As shown in Figure S7, dsRNAs were added to 48-well plates at a final concentration of 15 µg/ml. dsRNAs were tested in duplicate in independent plates. S2 cells were then seeded in the plates at a density of 5.0×10^5^ cells/well in 150 µl DS2-SFM medium. dsRNA-treated cells were incubated at 25°C for 1 day; 150 µl of fresh medium with 10% FBS was then added to each well to allow the cells to recover from dsRNA treatment. The dsRNA-treated cells were continuously incubated at 25°C for an additional three days to allow the knockdown of target gene expression. The efficiency of dsRNA mediated gene knock down was checked as previously described [41]. The treated cells (in 100 µl) together with 100 µl of fresh DS2-SFM medium were added into 48 well plates, and the treated cells were incubated overnight at 25°C before Cn infection. Cn strain H99 or AI100-dsRed was used to infect the dsRNA-treated S2 cells at an MOI of 5. At 3 h.p.i., the infected cells were washed 6∼8 times with 1×PBS and then 200 µl of fresh DS2-SFM medium supplemented with 30 µg/ml fluconazole was added to inhibit the replication of extracellular Cn cells, if any. To determine the number of Cn cells present in the medium at the starting time point (3 h.p.i., t_0_), the media were transferred to U-bottom 96-well plates (MicroTEST). Cn cells were centrifuged (1,200×g, 12 min) and resuspended in 200 µl of YPD broth. CFU assays were performed on the recovered Cn cells. To evaluate the effects of dsRNA-mediated knockdown of target genes in S2 cells on the phagocytosis of Cn cells, the infected cells were extensively washed and then lysed with 100 µl of 0.5% Tween 20 in sterile water for 15 min. 100 µl of YPD broth was added to the cell lysates. The material was thoroughly mixed and the cell lysate was transferred to a 96-well plate. CFU assays were performed as described above. To assess the effects of dsRNA treatment on Cn intracellular replication and escape at 24 h.p.i, media and cell lysates were first transferred to U-bottom 96-well plates. CFU assays were then performed on this material. Five µl of diluted cell lysate and Cn cell suspension were separately plated onto single well OmniTray (Nunc) plates containing YPD solid medium. CFUs were read after 24 hr of incubation at 30°C, and the infection index and RIF (see the *Mathematical Formulae* section) were calculated. dsRNAs that displayed a RIF that differed by more than 2-fold of SD from the untreated control in the screen were picked out for next round of screen. Candidates identified after two rounds of screening were selected for re-testing in triplicate.

### siRNA mediated depletion of target proteins in murine RAW264.7 macrophages

2.0×10^4^ RAW264.7 macrophages were seeded in each well of a 48-well plate one day before siRNA transfection. siRNA treatment was performed using Lipofectamine RNAiMAX or Lipofectamine 2000 transfection reagent (Invitrogen) in accordance with the manufacturer's instructions. All the siRNA constructs, including the scrambled siRNA and siRNAs targeting Atg2a, Atg5, Atg9a, Atg12 and LC3 were purchased from Santa Cruz Biotech., Inc. (Santa Cruz, CA, USA) or US Sigma-Aldrich. After two days of transfection, the medium was replaced with fresh DMED containing 10% FBS at least 2 hr before Cn (strain H99) infection. The growth and viability of siRNA-treated cells was monitored using the trypan blue exclusion assay [41].

### Immunoblotting analysis

At the indicated time points, siRNA transfected RAW264.7 macrophages or Cn infected host cells were washed three times with cold 1×PBS. The washed cells were then lysed without disrupting the intact Cn cells. ∼15 µg of the recovered protein extracts were separated by 12% SDS-PAGE, transferred onto polyvinylidene difluoride membranes (PVDM), and blotted with the indicated antibodies. The blots were detected using an enhanced chemiluminescence kit (Pierce, Rockford, IL). Blots were stripped and reprobed for the protein level of GAPDH (Glyceraldehyde 3-phosphate dehydrogenase, a constitutive housekeeping gene) as a loading control. Primary antibodies, including anti-LC3, anti-Atg5, anti-Atg12 and anti- GAPDH were purchased from Santa Cruz Biotech., Inc. Anti-Atg9a was purchased from Thermo Scientific. Densitometry of blots was performed using the ImageJ (http://rsbweb.nih.gov/ij/) software package. For each blot, the value of Blot area×Mean gray value×Integrated density (BaMI) was calculated. The ratio of blot LC3-II/LC3-I at an indicated time points were calculated as shown in the *Mathematical Formulae* section (see below).

### Mathematical formulae



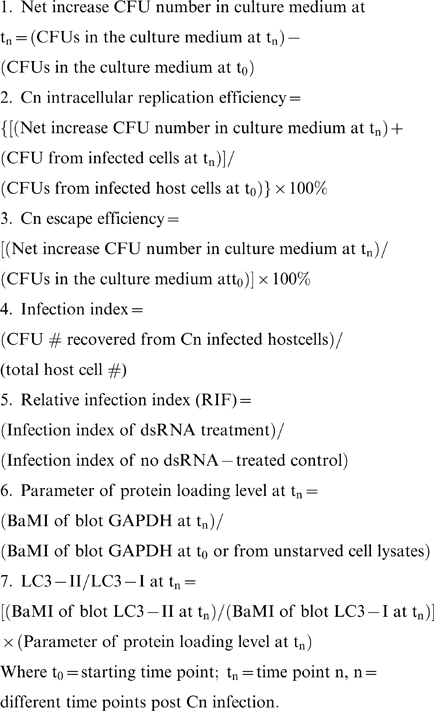



### Statistical analysis

All quantitative data were derived from results obtained from triplicate experiments that were independently performed at least three times. To easily compare results from independent experiments, the data from controls were normalized as 1 or 100%. The significance of the data was assessed using the Student's t-test.

### Accession numbers

The NCBI (http://www.ncbi.nlm.nih.gov/) database accession numbers for the mouse genes and gene products discussed in this paper are *Atg2a*: 329015, *Atg5*: 11793, *Atg9a*: 245860, *Atg12*: 67526 and *LC3β*: 67443.

## Supporting Information

Figure S1 *Cryptococcus neoformans* (Cn) infection of *Drosophila S2* cells. **A**. dsRed- or GFP-expression has no effect on Cn infection of S2 cells. *Drosophila* S2 cells were infected with the indicated Cn strains at an MOI of 5. At 3 h.p.i., the infected host cells were extensively washed and then continuously incubated in fresh medium at 28°C. At the indicated time points, the culture medium and the infected host cells were separately collected. CFU assays were then performed. The phagocytosis (3 h.p.i) and replication/escape (>12 h.p.i.) of Cn strains H99, AI100-dsRed and AI132-GFP in *Drosophila* S2 cells at 28°C were compared. No significant differences between the three Cn strains in phagocytosis or replication/escape were observed. Data represent the means ± standard deviations (SD) from one representative experiment of three total independent experiments performed. **B**. Phagocytosis of Cn cells by *Drosophila* S2 cells (upper panel) and Cn intracellular replication (lower panel). S2 cells were seeded on 12-mm coverslips placed on the bottom of a 24-well plate and infected with Cn strain AI100-dsRed (upper panel) or AI132-GFP (lower panel). At 1 or 3 h.p.i., the infected host cells were extensively washed and then continuously incubated at 28°C in fresh medium supplemented with 20 µg/ml fluconazole. At the indicated time points, the coverslips with infected S2 cells were fixed and stained with Alexa 488-conjugated phalloidin or rhodamine phalloidin to resolve the host cell actin cytoskeleton (lower panel). The infected cells were then visualized by fluorescence microscopy. Differential interference contrast (DIC) images of S2 cells (upper panel), cytoskeletal (rhodamine-phalloidin) staining of S2 cells (lower panel), dsRed- and GFP-labeleled Cn cells, and merged images are shown. Images were from one representative experiment of at least three total independent experiments.
(TIF)

Figure S2 The antifungal fluconazole has no cytotoxic effect on *Drsosphila* S2 cells. **A1**. Viability of S2 cells during a time course of coincubation with 40 µg/ml fluconazole. Medium with solvent alone (0.2% dimethyl sulfoxide: DMSO) and no fluconazole was used as control. At the indicated time points, the treated cells were stained with 0.2% trypan blue. The number of viable and non-viable cells was then counted under an inverted microscope, and the percentage of viable cells was calculated. To determine the viability of cells, at least 500 cells were counted from each well. Each treatment was performed in triplicate wells (24 well plate) in triplicate experiments. Data represent the means ± SD from three independent experiments. **A2**. Viability of S2 cells coincubated with fluconazole at the indicated concentrations (µg/ml) and time points. The culture medium with 0.2% DMSO (solvent alone) was used as the negative control. At the indicated time points, the viable cells were counted as described above. Data represent the means ± SD from at least three independent experiments. **B**. Dynamics of Cn cell growth in culture media containing fluconazole. Cn cells (AI100-dsRed, ∼1.5×10^4^) were inoculated in 200 µl of the indicated media containing 20 µg/ml fluconazole (in a 48-well plate format) and then incubated at 28°C (*Drosophila* S2 serum free medium: DS2-SFM) or at 37°C, 5% CO_2_ (DMEM+10% FBS). At the indicated time points post-inoculation, the culture media were collected and CFU assays were performed as described in the *Materials and Methods* section. Data represent the means ± SD from at least three independent experiments. *** indicates significance at p<0.001. **C**. Diagram of how Cn intracellular replication and escape from host cells in the presence of fluconazole was performed and monitored. Upon entry into host cells, Cn replicates in Cn containing vacuoles (CnCVs) and escapes *via* host cell lysis (A) and/or Cn extrusion (B). However, the population of extracellular Cn cells cannot replicate due to the presence of fluconazole. Cn cells present in the media and in host cells are separately collected and plated onto solid YPD media after serial dilution. The number of CFUs were determined after 24 hr of incubation at 30°C.
(TIF)

Figure S3 Antibodies against mammalian compartment marker proteins recognize orthologous proteins from *Drosophila* S2 cells (**A**) and display limited cross-reactivity with proteins from *Cryptococcus* cells (**B**). **A**. Antibodies directed against various markers of mammalian subcellular compartments recognize orthologous markers from S2 cells. Whole cell lysates from murine J774.A1 or RAW264.7 macrophages (MM) and *Drosophila* S2 (DS2) cells were analyzed by Western blot using antibodies directed against the indicated markers of mammalian organellar proteins. *Drosophila* orthologs of the expected sizes were specifically detected in these experiments. Comparison of the marker proteins from mouse (*Mus musculus*) and *Drosophila* orthologs is shown in the right panel **A**. The presented information about these proteins was garnered from the NCBI protein database (http://www.ncbi.nlm.nih.gov/protein/). **B**. No or limited cross-reactivity between Cn cells and the indicated antibodies against mammalian proteins was observed. J774.A1 cells were seeded onto 12-mm coverslips on the bottom of 24-well plates and then infected with Cn strain AI100-dsRed or H99 at an MOI of 3. At 1 or 3 h.p.i., , the host cells were washed one time with 1×PBS, and then fresh medium supplemented with 20 µg/ml fluconazole was added to each well. At different time points, coverslips were removed, fixed with 3.75% formaldehyde in 1×PBS, and prepared for fluorescence microscopy analysis. Immunofluorescence localization of different host proteins in host cells is shown in green and Cn cells are shown in red. Scale bar: 5 µM. The presented images were taken from a single representative experiment (n = 3) at the time point of 1 h.p.i.. Results from different time points and independent experiments were similar.
(TIF)

Figure S4 Cn cells interact with subcellular compartments in murine J774.A1 macrophages during a time course of infection. J774.A1 cells were seeded onto 12-mm coverslips on the bottom of 24-well plates and then infected with Cn strain AI100-dsRed at an MOI of 3. At 1 or 3 h.p.i., the host cells were washed three times with 1×PBS, and then fresh medium supplemented with 20 µg/ml fluconazole was added to each well. At the indicated time points, coverslips were removed, fixed with 3.75% formaldehyde in 1×PBS, and immunofluorescence staining using the indicated antibodies was performed. The percent of total infected cells analyzed in which the indicated marker was tightly associated with CnCvs was calculated and plotted as a function of time. Data represent the means ± SD from at least three independent experiments. For each compartment in each experiment, at least 500 internalized or replicative Cn cells were analyzed.
(TIF)

Figure S5 *Cryptococcus* strains from diverse genetic backgrounds behave similarly in *Drosophila* S2 and J774.A1 cells. Cn strains (H99, 87C, UA491, UA4223 and UM2) of assorted genetic backgrounds displayed similar internalization patterns in *Drosophila* S2 (**A1**) and J774.A1 (**B1**) cells. The indicated Cn strains displayed similar relative escape efficiencies (**A2** and **B2**) and intracellular replication efficiencies (**A3** and **B3**) in *Drosophila* S2 (**A**, leaf panel) and J774.A1 (**B**, right panel) cells. Relative escape and intracellular replication efficiencies were defined as described in the *Materials and Methods*. Data from the control (H99) were normalized to 100%, and all data represent the mean ± standard deviation from three independent experiments. *, **, *** indicates significance at P<0.05, P<0.01 and P<0.001, respectively.
(TIF)

Figure S6 Effects of selected drugs on *Cryptococcus* cell growth and infection. **A** and **B**. Effect of the indicated drugs on Cn (AI100-dsRed) replication in Insectagro DS2 serum free medium (DS2-SFM) at 28°C (**A**) and in DMEM with 10% FBS at 37°C, 5%CO_2_ (**B**) at the indicated concentration (Table S2) and time points. Cn growth in media supplemented with 0.2% dimethyl sulfoxide (DMSO) was used as control. **C**. Pre-treatment of Cn cells with the indicated drugs has no effect on the phagocytosis, escape and intracellular replication of the pathogen. Cn cells (H99) were incubated in DMEM with 10% FBS and the indicated drugs at the indicated concentration (Table S2). After 3 hr of incubation, the drugs were washed out. The treated Cn cells were re-suspended in fresh DMEM with 10% FBS and used as an inoculum for infecting host cells. The drug-treated and untreated Cn cells display similar levels of phagocytosis (C1), escape (C2) and intracellular replication efficiency (C3). All data represent the means ± standard deviations from three independent experiments.
(TIF)

Figure S7 Schema depicting the implemented RNAi screen for host factors that mediate *Cryptococcus* infection. *Drosophila* dsRNAs that target the knockdown of host factors are added to each well (**1**), 5.0×10^5^ S2 cells are then seeded into the plates containing dsRNAs and incubated at 25°C for 4 days (**2**). dsRNA-treated or untreated S2 cells are reseeded into ConA-treated plates after normalizing the cell number, The cells are allowed to attach for least 2 hrs (**3**). The dsRNA-treated and untreated S2 cells are then infected with Cn (AI100-dsRed or H99) (**4**). Wells containing infected cells are washed 6 times with 1×PBS at 3 h.p.i. (**5**). Fresh medium supplemented with 30 µg/ml fluconazole is added into each well and the infected cells are continuously incubated at 28°C for 24 hrs (**6**). To investigate the effects of dsRNA-treatment of S2 cells on Cn phagocytosis, at 3 h.p.i., fresh medium without any antifungal agent is added into each well after washing (6 times) with 1×PBS. The medium is then transferred to the wells of 96-well plates (**7**). The infected cells are lysed by incubation with 0.5% Tween 20 in sterile water for 10 to 15 min. The cell lysate is also transferred to the wells of 96-well plates (**7**). To evaluate the effects of dsRNA-treatment of S2 cells on Cn intracellular replication and escape, the media and cell lysates are also transferred to the wells of 96-well plates (**8**). After serial dilution, 5 µl of diluted medium or cell lysate is plated onto solid YPD medium. After 24 hr of incubation at 30°C, the number of CFUs in each sample is determined (**9**). After analyzing the data according to the descriptions in the *Materials and Methods*, candidate dsRNAs are subjected to a second round of screening. Confirmed hits (**10**) can then be validated in mammalian cells or in experimental animals (**11**). Finally, experiments that seek to elucidate the molecular and biochemical mechanisms of validated Cn host factors can be performed (**12**).
(TIF)

Figure S8 siRNA-mediated depletion of target proteins. **A**. Murine RAW264.7 macrophages were transfected with an Alexa-488 conjugated fluorescent scrambled siRNA (control). After 48 hrs of transfection, the cells were fixed, processed and analyzed for transfection efficiency by fluorescence microscopy. Scale bar: 10 µM. **B**. Depletion of host LC3 and Atg5 by siRNA treatment in RAW264.7 macrophages. To confirm reductions in the amounts of target proteins for siRNA treatment, siRNA transfected cells were harvested at the indicated time points and analyzed by Western blot using the indicated antibodies. Glyceraldehyde 3-phosphate dehydrogenase (GAPDH) was used as an internal loading control.
(TIF)

Table S1 *Cryptococcus neoformans* (Cn) strains used in this study.
(PDF)

Table S2 Pharmacological compounds used in this study.
(PDF)

Table S3 Effect of host factor depletion on *Crytpcoccus neformans* infection.
(PDF)

Supplemental Video 1 Early stages of Cn infection of S2 cells. Cn infection of *Drosophila* S2 cells during a 15 hrs period of infection. Acquisition time (at ∼3.5 h.p.i.) is shown in the upper right corner of the movie. Cn cells replicating within S2 cells are observed within the red-framed demarcations. Supplemental Video 1 can be found at: http://www.youtube.com/user/deFigueiredoLab.

Supplemental Video 2 Late stages of Cn infection of S2 cells. Cn infection of *Drosophila* S2 cells during a time period from ∼43 h.p.i. to 51 h.p.i.. Acquisition time (at ∼3.5 h.p.i.) is shown in the upper right corner of the movie. Phagocytosis of Cn cells, cell-to-cell spread of Cn cells, and extrusion of a Cn cell from the host can be seen within the yellow-framed demarcation. Supplemental Video 2 can be found at: http://www.youtube.com/user/deFigueiredoLab.
