# Supplementary figures and images for: Functional Analysis of Host Factors that Mediate the Intracellular Lifestyle of Cryptococcus neoformans
Source: PLoS Pathog. 2011 Jun 16;7(6):e1002078. doi: 10.1371/journal.ppat.1002078 (PMC3116820; doi:10.1371/journal.ppat.1002078)

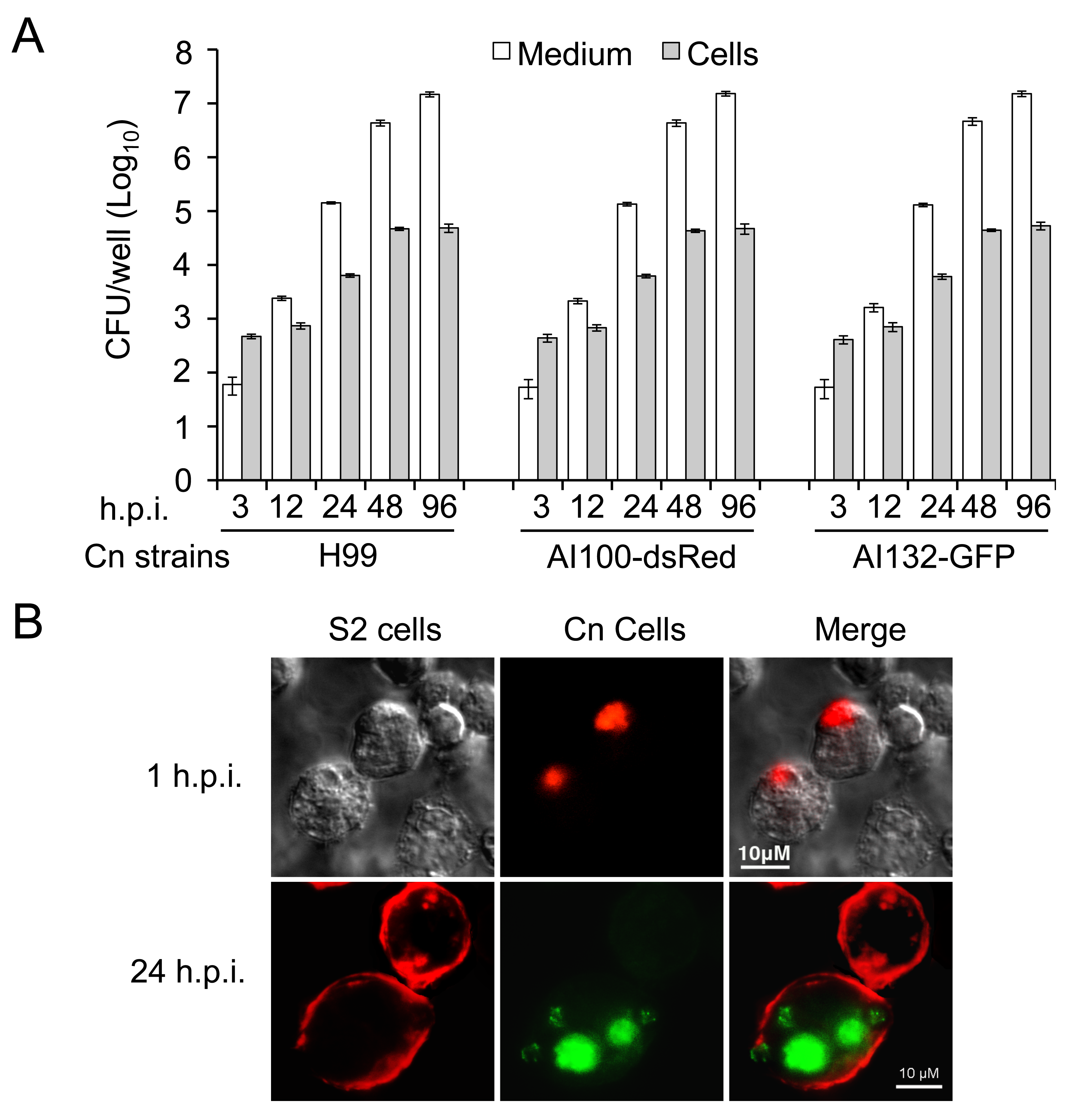

Supplement: Figure S1 — Cryptococcus neoformans (Cn) infection of Drosophila S2 cells. A. dsRed- or GFP-expression has no effect on Cn infection of S2 cells. Drosophila S2 cells were infected with the indicated Cn strains at an MOI of 5. At 3 h.p.i., the infected host cells were extensively washed and then continuously incubated in fresh medium at 28°C. At the indicated time points, the culture medium and the infected host cells were separately collected. CFU assays were then performed. The phagocytosis (3 h.p.i) and replication/escape (>12 h.p.i.) of Cn strains H99, AI100-dsRed and AI132-GFP in Drosophila S2 cells at 28°C were compared. No significant differences between the three Cn strains in phagocytosis or replication/escape were observed. Data represent the means ± standard deviations (SD) from one representative experiment of three total independent experiments performed. B. Phagocytosis of Cn cells by Drosophila S2 cells (upper panel) and Cn intracellular replication (lower panel). S2 cells were seeded on 12-mm coverslips placed on the bottom of a 24-well plate and infected with Cn strain AI100-dsRed (upper panel) or AI132-GFP (lower panel). At 1 or 3 h.p.i., the infected host cells were extensively washed and then continuously incubated at 28°C in fresh medium supplemented with 20 µg/ml fluconazole. At the indicated time points, the coverslips with infected S2 cells were fixed and stained with Alexa 488-conjugated phalloidin or rhodamine phalloidin to resolve the host cell actin cytoskeleton (lower panel). The infected cells were then visualized by fluorescence microscopy. Differential interference contrast (DIC) images of S2 cells (upper panel), cytoskeletal (rhodamine-phalloidin) staining of S2 cells (lower panel), dsRed- and GFP-labeleled Cn cells, and merged images are shown. Images were from one representative experiment of at least three total independent experiments. (TIF) [file ppat.1002078.s001.tif]

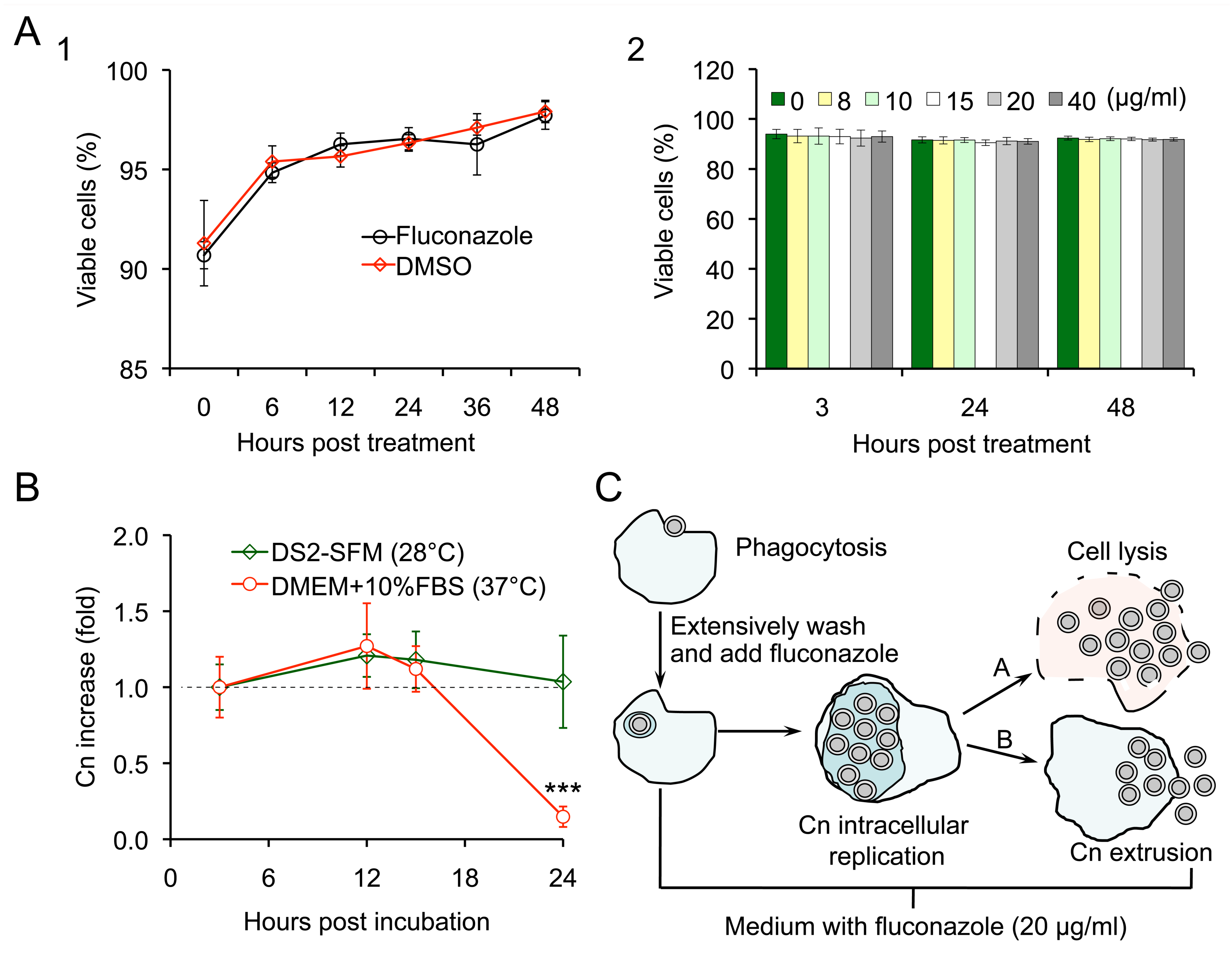

Supplement: Figure S2 — The antifungal fluconazole has no cytotoxic effect on Drsosphila S2 cells. A1. Viability of S2 cells during a time course of coincubation with 40 µg/ml fluconazole. Medium with solvent alone (0.2% dimethyl sulfoxide: DMSO) and no fluconazole was used as control. At the indicated time points, the treated cells were stained with 0.2% trypan blue. The number of viable and non-viable cells was then counted under an inverted microscope, and the percentage of viable cells was calculated. To determine the viability of cells, at least 500 cells were counted from each well. Each treatment was performed in triplicate wells (24 well plate) in triplicate experiments. Data represent the means ± SD from three independent experiments. A2. Viability of S2 cells coincubated with fluconazole at the indicated concentrations (µg/ml) and time points. The culture medium with 0.2% DMSO (solvent alone) was used as the negative control. At the indicated time points, the viable cells were counted as described above. Data represent the means ± SD from at least three independent experiments. B. Dynamics of Cn cell growth in culture media containing fluconazole. Cn cells (AI100-dsRed, ∼1.5×10^4^) were inoculated in 200 µl of the indicated media containing 20 µg/ml fluconazole (in a 48-well plate format) and then incubated at 28°C (Drosophila S2 serum free medium: DS2-SFM) or at 37°C, 5% CO2 (DMEM+10% FBS). At the indicated time points post-inoculation, the culture media were collected and CFU assays were performed as described in the Materials and Methods section. Data represent the means ± SD from at least three independent experiments. *** indicates significance at p<0.001. C. Diagram of how Cn intracellular replication and escape from host cells in the presence of fluconazole was performed and monitored. Upon entry into host cells, Cn replicates in Cn containing vacuoles (CnCVs) and escapes via host cell lysis (A) and/or Cn extrusion (B). However, the population of extracellular Cn cells canno [file ppat.1002078.s002.tif]

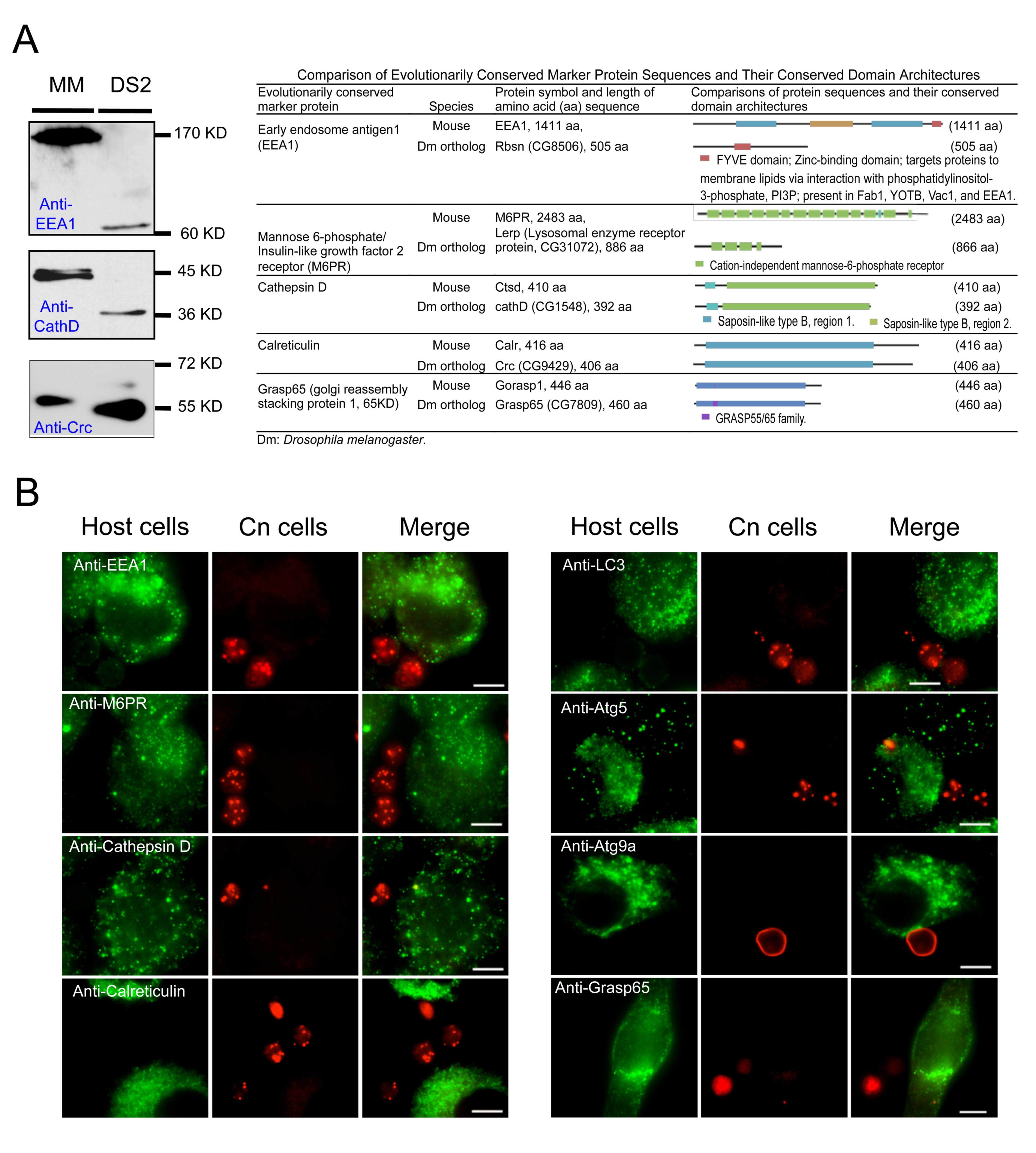

Supplement: Figure S3 — Antibodies against mammalian compartment marker proteins recognize orthologous proteins from Drosophila S2 cells (A) and display limited cross-reactivity with proteins from Cryptococcus cells (B). A. Antibodies directed against various markers of mammalian subcellular compartments recognize orthologous markers from S2 cells. Whole cell lysates from murine J774.A1 or RAW264.7 macrophages (MM) and Drosophila S2 (DS2) cells were analyzed by Western blot using antibodies directed against the indicated markers of mammalian organellar proteins. Drosophila orthologs of the expected sizes were specifically detected in these experiments. Comparison of the marker proteins from mouse (Mus musculus) and Drosophila orthologs is shown in the right panel A. The presented information about these proteins was garnered from the NCBI protein database (http://www.ncbi.nlm.nih.gov/protein/). B. No or limited cross-reactivity between Cn cells and the indicated antibodies against mammalian proteins was observed. J774.A1 cells were seeded onto 12-mm coverslips on the bottom of 24-well plates and then infected with Cn strain AI100-dsRed or H99 at an MOI of 3. At 1 or 3 h.p.i., , the host cells were washed one time with 1×PBS, and then fresh medium supplemented with 20 µg/ml fluconazole was added to each well. At different time points, coverslips were removed, fixed with 3.75% formaldehyde in 1×PBS, and prepared for fluorescence microscopy analysis. Immunofluorescence localization of different host proteins in host cells is shown in green and Cn cells are shown in red. Scale bar: 5 µM. The presented images were taken from a single representative experiment (n = 3) at the time point of 1 h.p.i.. Results from different time points and independent experiments were similar. (TIF) [file ppat.1002078.s003.tif]

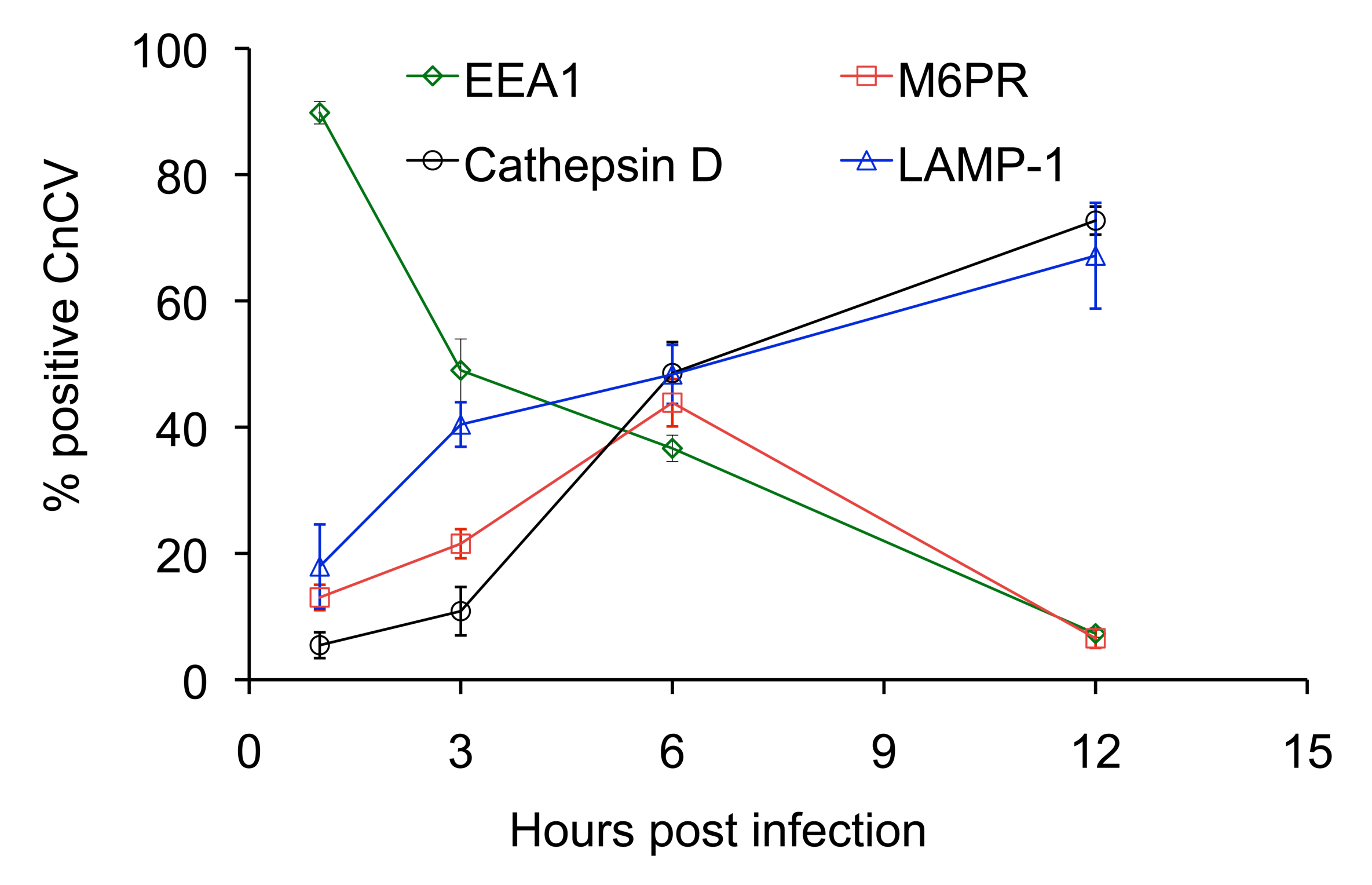

Supplement: Figure S4 — Cn cells interact with subcellular compartments in murine J774.A1 macrophages during a time course of infection. J774.A1 cells were seeded onto 12-mm coverslips on the bottom of 24-well plates and then infected with Cn strain AI100-dsRed at an MOI of 3. At 1 or 3 h.p.i., the host cells were washed three times with 1×PBS, and then fresh medium supplemented with 20 µg/ml fluconazole was added to each well. At the indicated time points, coverslips were removed, fixed with 3.75% formaldehyde in 1×PBS, and immunofluorescence staining using the indicated antibodies was performed. The percent of total infected cells analyzed in which the indicated marker was tightly associated with CnCvs was calculated and plotted as a function of time. Data represent the means ± SD from at least three independent experiments. For each compartment in each experiment, at least 500 internalized or replicative Cn cells were analyzed. (TIF) [file ppat.1002078.s004.tif]

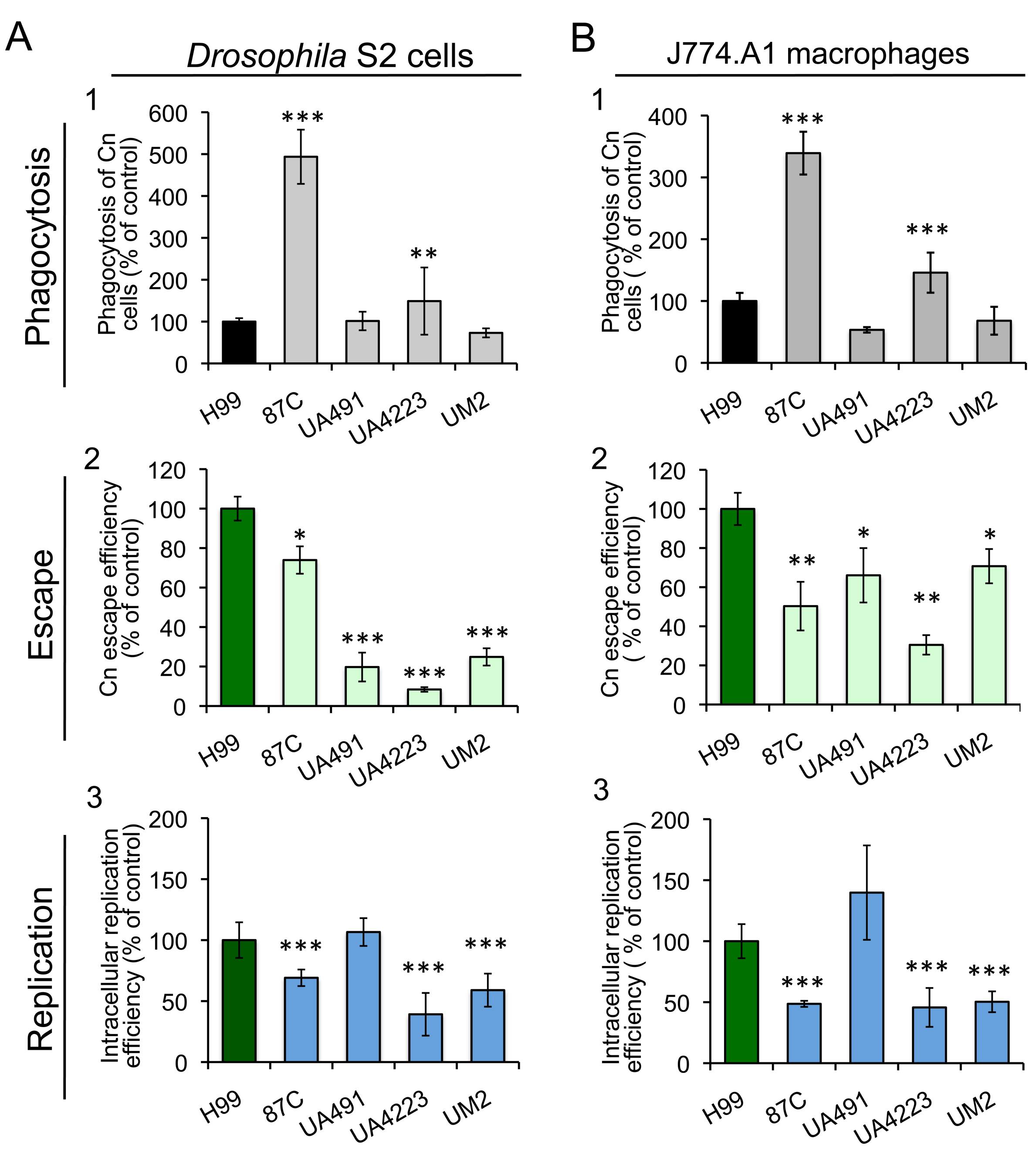

Supplement: Figure S5 — Cryptococcus strains from diverse genetic backgrounds behave similarly in Drosophila S2 and J774.A1 cells. Cn strains (H99, 87C, UA491, UA4223 and UM2) of assorted genetic backgrounds displayed similar internalization patterns in Drosophila S2 (A1) and J774.A1 (B1) cells. The indicated Cn strains displayed similar relative escape efficiencies (A2 and B2) and intracellular replication efficiencies (A3 and B3) in Drosophila S2 (A, leaf panel) and J774.A1 (B, right panel) cells. Relative escape and intracellular replication efficiencies were defined as described in the Materials and Methods . Data from the control (H99) were normalized to 100%, and all data represent the mean ± standard deviation from three independent experiments. *, **, *** indicates significance at P<0.05, P<0.01 and P<0.001, respectively. (TIF) [file ppat.1002078.s005.tif]

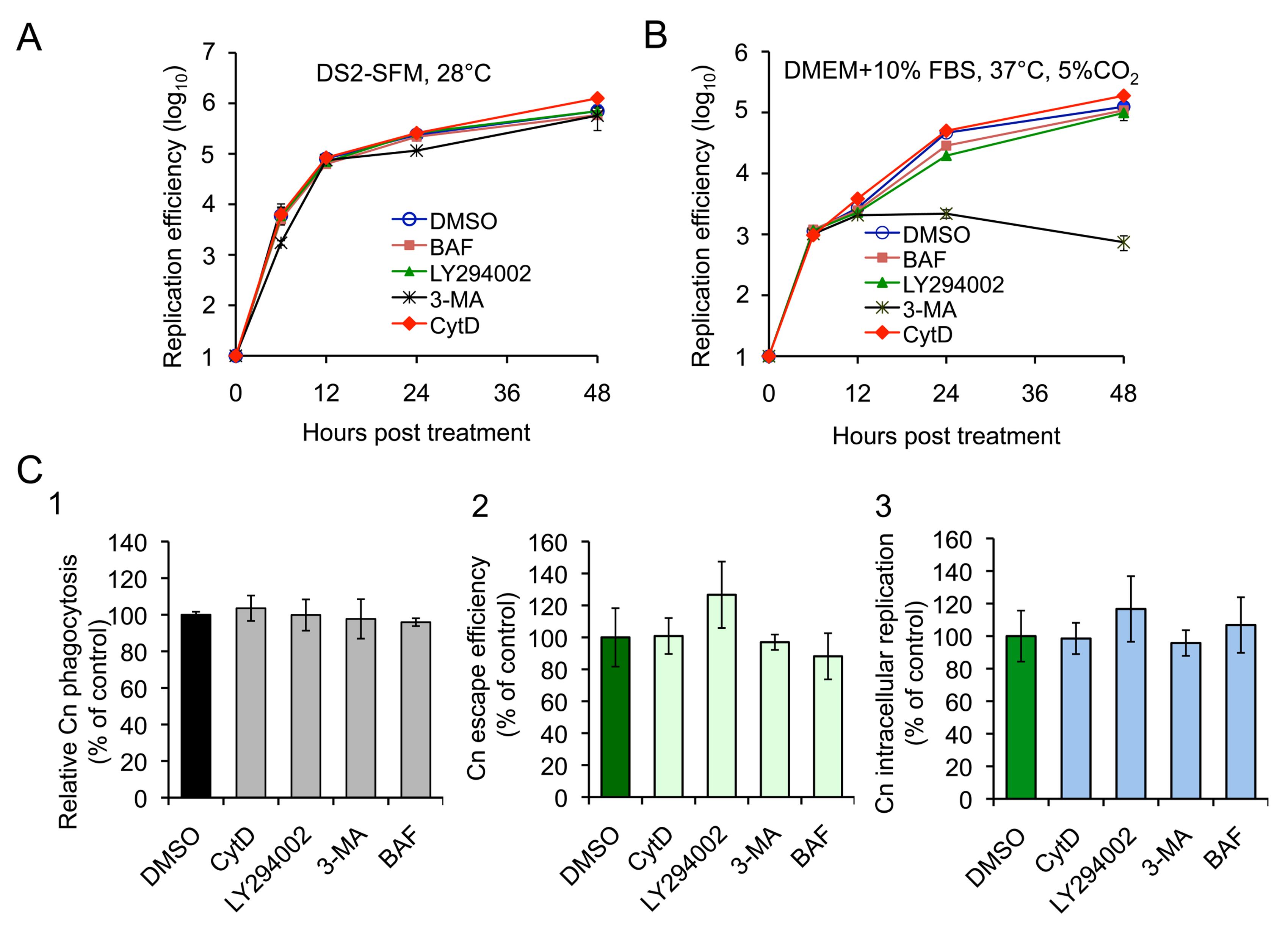

Supplement: Figure S6 — Effects of selected drugs on Cryptococcus cell growth and infection. A and B. Effect of the indicated drugs on Cn (AI100-dsRed) replication in Insectagro DS2 serum free medium (DS2-SFM) at 28°C (A) and in DMEM with 10% FBS at 37°C, 5%CO2 (B) at the indicated concentration (Table S2) and time points. Cn growth in media supplemented with 0.2% dimethyl sulfoxide (DMSO) was used as control. C. Pre-treatment of Cn cells with the indicated drugs has no effect on the phagocytosis, escape and intracellular replication of the pathogen. Cn cells (H99) were incubated in DMEM with 10% FBS and the indicated drugs at the indicated concentration (Table S2). After 3 hr of incubation, the drugs were washed out. The treated Cn cells were re-suspended in fresh DMEM with 10% FBS and used as an inoculum for infecting host cells. The drug-treated and untreated Cn cells display similar levels of phagocytosis (C1), escape (C2) and intracellular replication efficiency (C3). All data represent the means ± standard deviations from three independent experiments. (TIF) [file ppat.1002078.s006.tif]

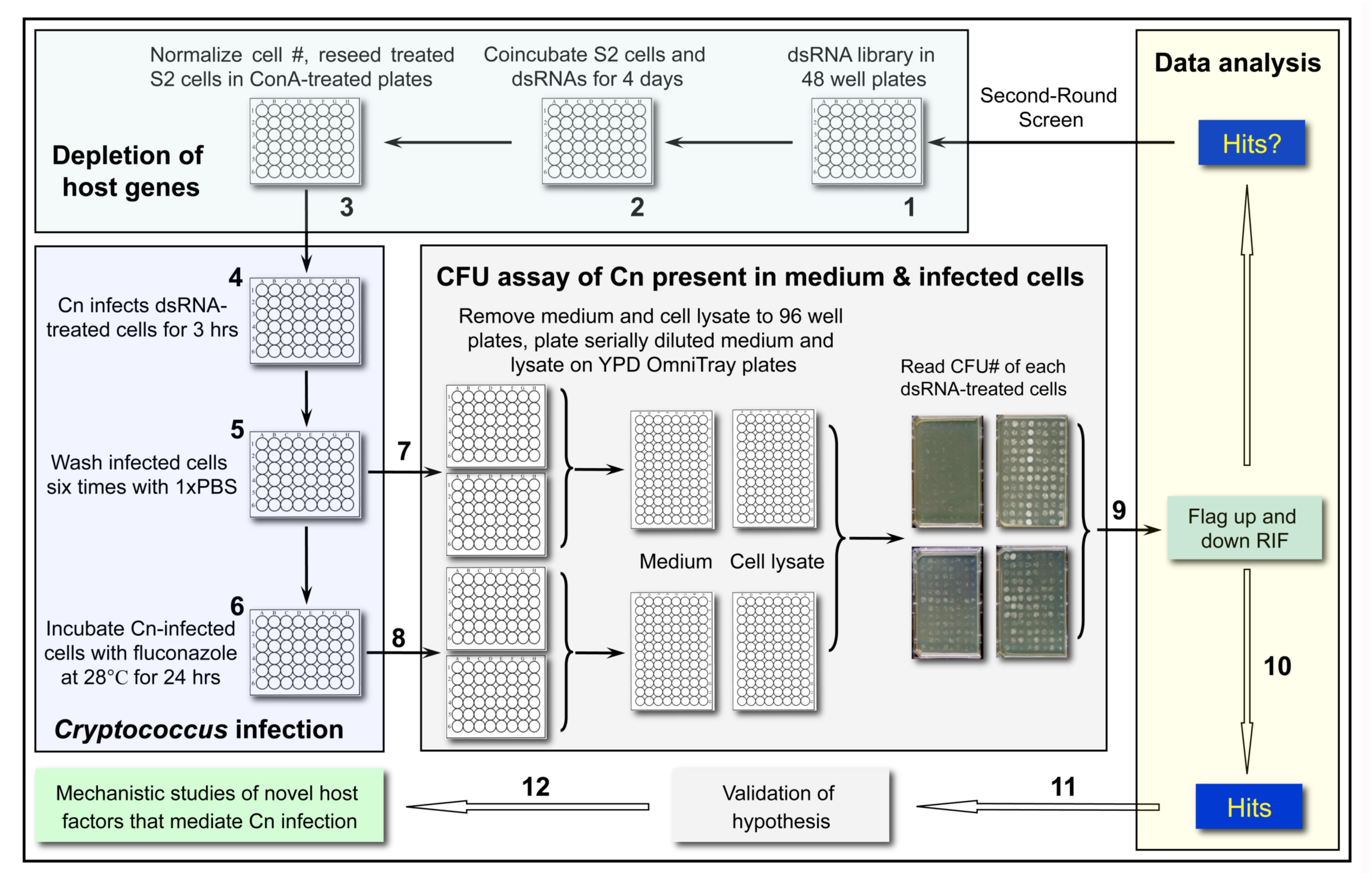

Supplement: Figure S7 — Schema depicting the implemented RNAi screen for host factors that mediate Cryptococcus infection. Drosophila dsRNAs that target the knockdown of host factors are added to each well (1), 5.0×10^5^ S2 cells are then seeded into the plates containing dsRNAs and incubated at 25°C for 4 days (2). dsRNA-treated or untreated S2 cells are reseeded into ConA-treated plates after normalizing the cell number, The cells are allowed to attach for least 2 hrs (3). The dsRNA-treated and untreated S2 cells are then infected with Cn (AI100-dsRed or H99) (4). Wells containing infected cells are washed 6 times with 1×PBS at 3 h.p.i. (5). Fresh medium supplemented with 30 µg/ml fluconazole is added into each well and the infected cells are continuously incubated at 28°C for 24 hrs (6). To investigate the effects of dsRNA-treatment of S2 cells on Cn phagocytosis, at 3 h.p.i., fresh medium without any antifungal agent is added into each well after washing (6 times) with 1×PBS. The medium is then transferred to the wells of 96-well plates (7). The infected cells are lysed by incubation with 0.5% Tween 20 in sterile water for 10 to 15 min. The cell lysate is also transferred to the wells of 96-well plates (7). To evaluate the effects of dsRNA-treatment of S2 cells on Cn intracellular replication and escape, the media and cell lysates are also transferred to the wells of 96-well plates (8). After serial dilution, 5 µl of diluted medium or cell lysate is plated onto solid YPD medium. After 24 hr of incubation at 30°C, the number of CFUs in each sample is determined (9). After analyzing the data according to the descriptions in the Materials and Methods , candidate dsRNAs are subjected to a second round of screening. Confirmed hits (10) can then be validated in mammalian cells or in experimental animals (11). Finally, experiments that seek to elucidate the molecular and biochemical mechanisms of validated Cn host factors can be performed (12). (TIF) [file ppat.1002078.s007.tif]

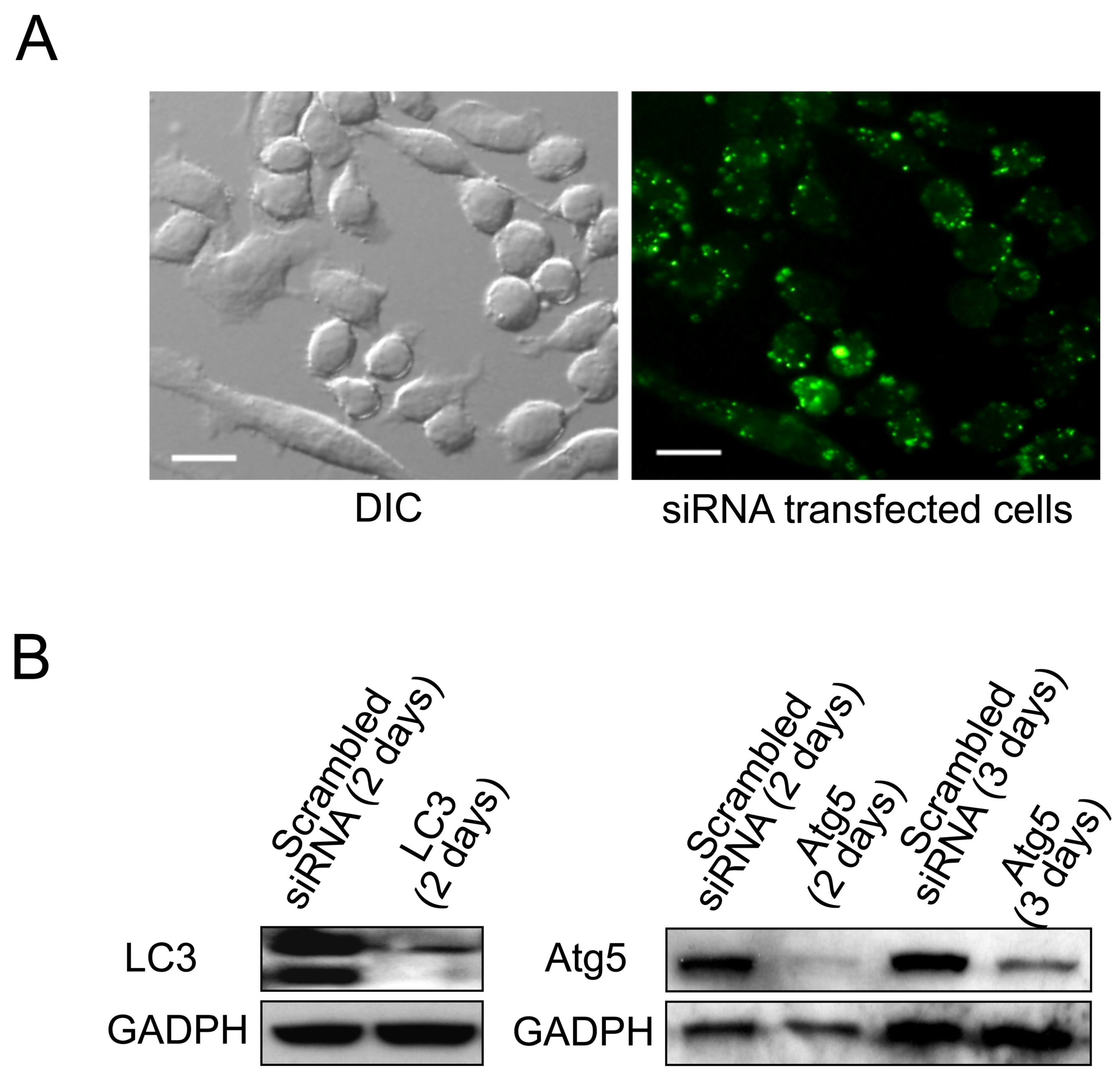

Supplement: Figure S8 — siRNA-mediated depletion of target proteins. A. Murine RAW264.7 macrophages were transfected with an Alexa-488 conjugated fluorescent scrambled siRNA (control). After 48 hrs of transfection, the cells were fixed, processed and analyzed for transfection efficiency by fluorescence microscopy. Scale bar: 10 µM. B. Depletion of host LC3 and Atg5 by siRNA treatment in RAW264.7 macrophages. To confirm reductions in the amounts of target proteins for siRNA treatment, siRNA transfected cells were harvested at the indicated time points and analyzed by Western blot using the indicated antibodies. Glyceraldehyde 3-phosphate dehydrogenase (GAPDH) was used as an internal loading control. (TIF) [file ppat.1002078.s008.tif]
