# Supplementary material for: Functional Analysis of Host Factors that Mediate the Intracellular Lifestyle of Cryptococcus neoformans
Source: PLoS Pathog. 2011 Jun 16;7(6):e1002078. doi: 10.1371/journal.ppat.1002078 (PMC3116820; doi:10.1371/journal.ppat.1002078)
Supplement: Table S1 — Cryptococcus neoformans (Cn) strains used in this study. (PDF) [file ppat.1002078.s009.pdf]

Table S1. *Cryptococcus neoformans* (Cn) strains used in this study

Cn strains	Genotype	Strain Characteristics	References
H99	Wild type	Cn var. *grubii*, wild-type strain, highly virulent in mice	[1]
AI100-dsRed	P_histoneH3-DsRed-SKL-T_hH3-NEO	H99 with an ectopic integration of DsRed.	[2]
AI132-GFP	P_histoneH3-HEM15-GFP-NAT	H99 with an ectopic integration of GFP tagged after the *HEM15* protein.	[2]
Cap59	Cap59::HYG	Mutant in H99 background, avirulent, no capsule	[3,4]
XX44	Scs7::NEO	Mutant in H99 background, deletion of the gene encoding the sphingolipid alpha-hydroxylase. Virulence in animals not tested, but likely dramatically reduced as the mutant displays defective growth in 5% CO_2.	Xie X and Lin X, unpublished data
HMC1	lac1::HYG	Mutant in H99 background, modestly reduced in virulence, defective in melanin production	[5,6,7]
MCD16	lac1 mutant	Mutant in H99 background, modestly reduced in virulence, defective in melanin production	[5,6,7]
AI81	bwc1::NAT	Mutant in H99 background, modestly reduced in virulence, required for light sensing.	[8]
XL1601	Znf2::NEO	Mutant in H99 background, modestly enhanced in virulence. Cells are locked in yeast phase and cannot undergo dimorphic switch to hyphal growth during mating	[9]
87C	Wild type	Cn var. *grubii*	[10]
UA491	Wild type	Cn var. *neoformans*	[11]
UA4223	Wild type	Cn var. *neoformans*	[11]
UM2	Wild type	Hybrid of Cn var. *grubii* and Cn var. *neoformans* (A/D strains)	[10]

References

1. Perfect JR, Lang SD, Durack DT (1980) Chronic cryptococcal meningitis: a new experimental model in rabbits. Am J Pathol 101: 177-194.
2. Idnurm A, Giles SS, Perfect JR, Heitman J (2007) Peroxisome function regulates growth on glucose in the basidiomycete fungus *Cryptococcus neoformans*. Eukaryot Cell 6: 60-72.
3. Geunes-Boyer S, Oliver TN, Janbon G, Lodge JK, Heitman J, et al. (2009) Surfactant protein D increases phagocytosis of hypocapsular Cryptococcus neoformans by murine macrophages and enhances fungal survival. Infect Immun 77: 2783-2794.
4. Nelson RT, Pryor BA, Lodge JK (2003) Sequence length required for homologous recombination in *Cryptococcus neoformans*. Fungal Genet Biol 38: 1-9.
5. Pukkila-Worley R, Gerrald QD, Kraus PR, Boily MJ, Davis MJ, et al. (2005) Transcriptional network of multiple capsule and melanin genes governed by the *Cryptococcus neoformans* cyclic AMP cascade. Eukaryot Cell 4: 190-201.
6. Zhu X, Williamson PR (2004) Role of laccase in the biology and virulence of *Cryptococcus neoformans*. FEMS Yeast Res 5: 1-10.
7. Salas SD, Bennett JE, Kwon-Chung KJ, Perfect JR, Williamson PR (1996) Effect of the laccase gene CNLAC1, on virulence of *Cryptococcus neoformans*. J Exp Med 184: 377-386.
8. Idnurm A, Heitman J (2005) Light controls growth and development via a conserved pathway in the fungal kingdom. PLoS Biol 3: e95.
9. Lin X, Jackson JC, Feretzaki M, Xue C, Heitman J (2010) Transcription factors Mat2 and Znf2 operate cellular circuits orchestrating opposite- and same-sex mating in Cryptococcus neoformans. PLoS Genet 6: e1000953.
10. Chaturvedi V, Fan J, Stein B, Behr MJ, Samsonoff WA, et al. (2002) Molecular genetic analyses of mating pheromones reveal intervariety mating or hybridization in *Cryptococcus neoformans*. Infect Immun 70: 5225-5235.
11. Barchiesi F, Cogliati M, Esposto MC, Spreghini E, Schimizzi AM, et al. (2005) Comparative analysis of pathogenicity of *Cryptococcus neoformans* serotypes A, D and AD in murine cryptococcosis. J Infect 51: 10-16.
