# Supplementary material for: Functional Analysis of Host Factors that Mediate the Intracellular Lifestyle of Cryptococcus neoformans
Source: PLoS Pathog. 2011 Jun 16;7(6):e1002078. doi: 10.1371/journal.ppat.1002078 (PMC3116820; doi:10.1371/journal.ppat.1002078)
Supplement: Table S2 — Pharmacological compounds used in this study. (PDF) [file ppat.1002078.s010.pdf]

Table S2. Pharmacological compounds used in this study

Drug Name	Function	Concentration	References
Cytochalasin D (CytD)	Inhibitor of actin polymerization (disruption of actin microfilaments and activation of p53-dependent pathways which results in the arrest of the cell cycle at the G1-S transition)	2.5 μg/ml	[1]
LY294002	PI3 kinase inhibitor (blocks PI3 kinase-dependent Akt phosphorylation and kinase activity)	30 μM	[2]
3-Methyladenine (3-MA)	Autophagy Inhibitor; PI3 kinase inhibitor	10 mM	[3]
Bafilomycin A1 (BAF)	Inhibitor of the vacuolar type H(+)-ATPase and endosomal acidification	100 nM	[4]

References

1. Qin QM, Pei J, Ancona V, Shaw BD, Ficht TA, et al. (2008) RNAi screen of endoplasmic reticulum-associated host factors reveals a role for IRE1alpha in supporting Brucella replication. PLoS Pathog 4: e1000110.
2. Blommaart EF, Krause U, Schellens JP, Vreeling-Sindelarova H, Meijer AJ (1997) The phosphatidylinositol 3-kinase inhibitors wortmannin and LY294002 inhibit autophagy in isolated rat hepatocytes. Eur J Biochem 243: 240-246.
3. Petiot A, Ogier-Denis E, Blommaart EF, Meijer AJ, Codogno P (2000) Distinct classes of phosphatidylinositol 3'-kinases are involved in signaling pathways that control macroautophagy in HT-29 cells. J Biol Chem 275: 992-998.
4. Yamamoto A, Tagawa Y, Yoshimori T, Moriyama Y, Masaki R, et al. (1998) Bafilomycin A1 prevents maturation of autophagic vacuoles by inhibiting fusion between autophagosomes and lysosomes in rat hepatoma cell line, H-4-II-E cells. Cell Struct Funct 23: 33-42.
