# Supplementary material for: Functional Analysis of Host Factors that Mediate the Intracellular Lifestyle of Cryptococcus neoformans
Source: PLoS Pathog. 2011 Jun 16;7(6):e1002078. doi: 10.1371/journal.ppat.1002078 (PMC3116820; doi:10.1371/journal.ppat.1002078)
Supplement: Table S3 — Effect of host factor depletion on Crytpcoccus neformans infection. (PDF) [file ppat.1002078.s011.pdf]

Table S3. Effect of host factor depletion on *Crytpcoccus neformans* infection

Symbol	CG #	Function[a]	Relative infection index				Play role in regulation of the infection by other pathogens[c]	Human homolog
			3 h.p.i.		24 h.p.i.			
		**Reduced phagocytosis**	RIF[b]	SD	RIF	SD		
Rab11	CG5771	Small GTPase mediated signal transduction, regulation of transcription	0.50	0.18	0.63	0.19	Cc[c], Lm[d], Mf[e]	RAB11A
Rab23	CG2108	Small GTPase mediated signal transduction, protein transport	0.48	0.24	0.77	0.01		RAB23
CG17565	CG17565	Rab-protein geranylgeranyltransferase complex	0.50	0.32	0.93	0.48		FNTB
Eip93F	CG18389	Phagocytosis, engulfment	0.43	0.18	0.70	0.11		?
Hr38	CG1864	Hormone receptor transcription factor	0.47	0.19	0.80	0.29	Ca[f]	NR4A2
CG7033	CG7033	Vhaperonin-containing T-complex	0.45	0.18	0.55	0.19		CCT2
CG2158	CG2158	Nuclear protein import/export	0.49	0.18	0.57	0.16	Ca	NUP50
TER94	CG2331	ER membrane fusion, microtubule cytoskeleton biogenesis	0.33	0.14	1.30	1.29	Ct[g], Bm[h], Lm, Mf	VCP
lap	CG2520	Receptor-mediated endocytosis	0.49	0.14	0.53	0.06		PICALM
Ost48	CG9022	Protein amino acid N-linked glycosylation	0.46	0.13	0.54	0.33		DDOST
exo84	CG6095	Vesicle-mediated transport, exocyst	0.30	0.18	0.74	0.33		EXOC8
sec8	CG2095	Vesicle docking during exocytosis, exocyst	0.29	0.12	0.51	0.19		EXOC4
sec10	CG6159	Vesicle-mediated transport, exocytosis	0.33	0.22	0.67	0.64		EXOC5
Vha14	CG8210	Vacuolar proton-transporting V-type ATPase,	0.25	0.26	0.82	1.37		ATP6V1F
αTub84B	CG1913	Microtubule-based movement, protein polymerization, GTPase activity	0.37	0.04	0.58	0.35	Lm, Mf	TUBA1A
δCOP	CG14813	COPI vesicles, endocytosis	0.23	0.07	0.56	0.39	Lm, Mf, Ca	ARCN1
		**Reduced replication/escape**						
CG14870	CG14870	Cilium assembly	0.66	0.46	0.37	0.20		B9D1
CG1126	CG1126	Cilium assembly	0.64	0.46	0.44	0.26		BBS5
Pk61C	CG1210	Plasma membrane, protein kinase activity, ATP binding	0.68	0.57	0.46	0.24		PDPK1
phl	CG2845	Protein kinase activity, amino acid phosphorylation	0.52	0.28	0.43	0.21		BRAF
Orct	CG6331	Apoptosis, organic cation transport	0.72	0.22	0.42	0.09		SLC22A13
Aats-gly	CG6778	Glycyl-tRNA aminoacylation	0.68	0.38	0.41	0.09		GARS
exo70	CG7127	Synaptic vesicle docking during exocytosis	0.82	0.47	0.48	0.14		EXOC7
Apc	CG1451	Microtubule associated complex	0.62	0.21	0.44	0.25		APC
Myo31DF	CG7438	Actin binding; ATPase activity, myosin complex	0.53	0.56	0.35	0.27	Ct	MYO1D
ERp60	CG8983	Protein folding; cell redox homeostasis	0.85	0.51	0.43	0.22		PDIA3

Rac1	CG2248	JNK cascade signaling, cytoskeletal rearrangements	1.08	0.78	0.36	0.26	Ct, Bm, Ca	RAC2
sec5	CG8843	Neurotransmitter secretion; synaptic vesicle docking during exocytosis	0.67	0.58	0.27	0.18	Lm, Mf	SEC5L1
Crc	CG9429	Calcium ion/unfolded protein binding,nervous system development	0.63	0.28	0.45	0.10	Bm	CALR
Atg2	CG1241	Autophagy	0.51	0.43	0.50	0.07		ATG2A
Atg5	CG1643	Autophagy	0.58	0.08	0.43	0.03		ATG5
Atg9	CG3615	Autophagy	1.03	0.04	0.50	0.15		ATG9A
		**Reduced both phagocytosis and replication/escape**						
Rheb	CG1081	Response to starvation, small GTPase mediated signal transduction	0.20	0.19	0.16	0.02		RHEB
Rab5	CG3664	Early endosome; GTPase activity; GTP binding	0.36	0.13	0.30	0.22	Cc, Lm, Mf	RAB5A
Act42A	CG12051	Actin, cytoskeleton	0.29	0.18	0.11	0.11	Mf, Ca	ACTG1
Act5C	CG4027	Cytoskeleton organization, actin filament	0.24	0.19	0.18	0.01	Cc, Lm, Mf , Ca	ACTG1
Act79B	CG7478	Cytoskeleton organization, actin filament	0.15	0.21	0.11	0.19	Cc, Ct	ACTG2
Act88F	CG5178	Cytoskeleton organization, actin filament	0.20	0.12	0.22	0.09	Ca	?
βTub56D	CG9277	Cytoskeleton; microtubule-based movement, protein polymerization	0.41	0.18	0.33	0.18	Lm, Mf	?
polo	CG12306	Protein serine/threonine kinase activity, phosphorylates APC/C	0.35	0.16	0.35	0.34	Ca	PLK1
CG5830	CG5830	Phosphatase activity	0.21	0.10	0.49	0.19		CTDSP1
Pi3K59F	CG5373	phosphoinositide 3-kinase complex, class III	0.51	0.35	0.47	0.22	Bm	PIK3C3
dl	CG6667	Regulation of transcription, Toll signaling pathway	0.27	0.01	0.24	0.22		REL
ftz-f1	CG4059	Salivary gland cell autophagic cell death, regulation of transcription	0.31	0.06	0.31	0.37		NR5A2
CG6842	CG6842	peptide deformylase, protein modification	0.15	0.05	0.37	0.14	Lm, Ca	VPS4B
th	CG12284	Ubiquitin-protein ligase activity, anti-apoptosis	0.10	0.10	0.32	0.38	Cc, Mf	?
CanA1	CG1455	Vesicle-mediated transport; calcineurin complex,	0.09	0.00	0.24	0.16	Ct	PPP3CA
Gdi	CG4422	Vesicle-mediated transport, Rab GDP-dissociation inhibitor activity	0.36	0.25	0.40	0.05	Lm	GDI2
Vha13	CG6213	Vacuolar proton-transporting V-type ATPase complex	0.18	0.19	0.16	0.17	Lm	ATP6V1G1
Vha44	CG8048	Vacuolar proton-transporting V-type ATPase, V1 domain	0.26	0.30	0.19	0.11	Lm	ATP6V1C1
sec15	CG7034	Vesicle-mediated transport, synaptic vesicle docking during exocytosis	0.34	0.19	0.39	0.19		EXOC6
sec23	CG1250	ER to Golgi transport vesicle, COPII vesicle coat	0.24	0.07	0.33	0.16	Bm, Lm, Mf	SEC23A
αCop	CG7961	Vesicle-mediated transport, COPI vesicles coat	0.21	0.10	0.08	0.03	Cc, Lm, Mf, Ca	COPA
ATPsyn-γ	CG7610	ATP synthesis coupled proton transport, ATPase	0.43	0.20	0.32	0.11	Ca	ATP5C1
MEK	CG15793	MAPKKK cascade	0.23	0.04	0.38	0.20		MAP2K1
Tsc1	CG6147	Regulation of cell growth	0.46	0.28	0.22	0.07	Cc	TSC1
		**Enhanced replication/escape**						
Hsp83	CG1242	Response to stress, actin filament-based process	1.99	1.02	4.22	2.12		HSP90AA2

[a]Genes were categorized according to the Gene Ontology (GO) index, including GO molecular function, cellular component, or protein domains as reported in FlyBase (http://flybase.org/). [b]Relative infection index [RIF, RIF=[(Infection index of dsRNA treatment)/(Infection index of no dsRNA-treated control); where infection index= (CFU # recovered from Cn infected host cells)/(total host cell #)] was determined as described in the *Materials and Methods*. *Drosophila* S2 cells were treated with dsRNAs for 4 days before *Cryptococcus neoformans* (AI100-dsRed or H99) infection. All data represent the means ± standard deviations (SD) from at least three independent screens. The average RIF of the control that was not treated with RNAi was regarded as 1 (1.00±0.249). dsRNAs that gave RIF ≤ (1.00-2 ×SD, 0.50) or ≥ (1.00+2×SD, 1.50) were regarded as hits. [c]*Cc: Chlamydia caviae* [1], [d]*Lm: Listeria monocytogenes* [2,3], [e]Mf: *Mycobacterium fortuitum* [2,4]. [f]Ca: *Candida albicans* [5]. [g]Ct: *Chlamydia trachomatis* [6]. [h]Bm: *Brucella melitensis* [7].

References

1. Derre I, Pypaert M, Dautry-Varsat A, Agaisse H (2007) RNAi screen in *Drosophila* cells reveals the involvement of the Tom complex in Chlamydia infection. PLoS Pathog 3: 1446-1458.
2. Agaisse H, Burrack LS, Philips JA, Rubin EJ, Perrimon N, et al. (2005) Genome-wide RNAi screen for host factors required for intracellular bacterial infection. Science 309: 1248-1251.
3. Cheng LW, Viala JP, Stuurman N, Wiedemann U, Vale RD, et al. (2005) Use of RNA interference in *Drosophila* S2 cells to identify host pathways controlling compartmentalization of an intracellular pathogen. Proc Natl Acad Sci U S A 102: 13646-13651.
4. Philips JA, Rubin EJ, Perrimon N (2005) *Drosophila* RNAi screen reveals CD36 family member required for mycobacterial infection. Science 309: 1251-1253.
5. Stroschein-Stevenson SL, Foley E, O'Farrell PH, Johnson AD (2006) Identification of *Drosophila* gene products required for phagocytosis of *Candida albicans*. PLoS Biol 4: e4.
6. Elwell CA, Ceesay A, Kim JH, Kalman D, Engel JN (2008) RNA interference screen identifies Abl kinase and PDGFR signaling in *Chlamydia trachomatis* entry. PLoS Pathog 4: e1000021.
7. Qin QM, Pei J, Ancona V, Shaw BD, Ficht TA, et al. (2008) RNAi screen of endoplasmic reticulum-associated host factors reveals a role for IRE1alpha in supporting *Brucella* replication. PLoS Pathog 4: e1000110.
