# Supplementary material for: Functional Analysis of Host Factors that Mediate the Intracellular Lifestyle of Cryptococcus neoformans
Source: PLoS Pathog. 2011 Jun 16;7(6):e1002078. doi: 10.1371/journal.ppat.1002078 (PMC3116820; doi:10.1371/journal.ppat.1002078)
Supplement: Supplemental Video 1 — Early stages of Cn infection of S2 cells. Cn infection of Drosophila S2 cells during a 15 hrs period of infection. Acquisition time (at ∼3.5 h.p.i.) is shown in the upper right corner of the movie. Cn cells replicating within S2 cells are observed within the red-framed demarcations. Supplemental Video 1 can be found at: http://www.youtube.com/user/deFigueiredoLab. [file ppat.1002078.s012.pdf]

Supplemental Video 1: Early stages of Cn infection of *Drosophila* S2 cells
http://www.youtube.com/user/deFigueiredoLab?blend=1&ob=5#p/u/1/F9aSd0p2xHE
