# Supplementary material for: Functional Analysis of Host Factors that Mediate the Intracellular Lifestyle of Cryptococcus neoformans
Source: PLoS Pathog. 2011 Jun 16;7(6):e1002078. doi: 10.1371/journal.ppat.1002078 (PMC3116820; doi:10.1371/journal.ppat.1002078)
Supplement: Supplemental Video 2 — Late stages of Cn infection of S2 cells. Cn infection of Drosophila S2 cells during a time period from ∼43 h.p.i. to 51 h.p.i.. Acquisition time (at ∼3.5 h.p.i.) is shown in the upper right corner of the movie. Phagocytosis of Cn cells, cell-to-cell spread of Cn cells, and extrusion of a Cn cell from the host can be seen within the yellow-framed demarcation. Supplemental Video 2 can be found at: http://www.youtube.com/user/deFigueiredoLab. [file ppat.1002078.s013.pdf]

Supplemental Video 2: Late stages of Cn infection of *Drosophila* S2 cells
http://www.youtube.com/user/deFigueiredoLab?blend=1&ob=5#p/u/0/9XdxN40MApE
